# Supplementary material for: The pan-genome and local adaptation of Arabidopsis thaliana
Source: Nat Commun. 2023 Oct 6;14:6259. doi: 10.1038/s41467-023-42029-4 (PMC10558531; doi:10.1038/s41467-023-42029-4)
Supplement: Supplementary file 1 — Supplementary Information [file 41467_2023_42029_MOESM1_ESM.pdf]

The pan-genome and local adaptation of *Arabidopsis thaliana*

Kang *et al.*

Supplementary Table 1. Summary of the 32 *Arabidopsis thaliana* ecotypes in this study.

	Name	CS Number	Country	Latitude	Longitude	Altitude
Non-Relict	Yilong-0	CS79063	China	31.56402	106.7062	506
	Bor-1	CS22590	Czech Republic	49.4013	16.2326	697
	Cdm-0	CS76410	Spain	39.73	-5.74	157
	Got-22	CS22609	Germany	51.5338	9.9355	310
	Kondara	CS22651	Tajikistan	38.48	68.49	641
	Kz-9	CS22607	Kazakhstan	49.5	73.1	283
	LL-0	CS22650	Spain	41.59	2.49	329
	Mammo-1	CS76365	Italy	38.36	16.23	146
	Ms-0	CS22655	Russian Federation	55.7522	37.6322	2,306
	Pra-6	CS76416	Spain	41.05	-3.54	440
	Pu2-23	CS22593	Czech Republic	49.42	16.36	2,698
	Sij-1	CS76379	Uzbekistan	41.45	70.05	666
	Sorbo	CS22653	Tajikistan	38.35	68.48	350
	TueSB30-3	CS76403	Germany	48.53	9.06	165
	Hs-0	CS76145	Germany	52.24	9.44	42
	LI-OF-095	CS76165	United States of America	40.9447	-72.8615	148
	Per-1	CS76210	Russian Federation	58	56.3167	148
	St-0	CS76231	Sweden	59	18	25
	Kelsterbach-2	CS28382	Germany	50.0667	8.5333	93
	Nz-1	CS28578	New Zealand	-37.7871	175.283	48
	Dra-2	CS28214	Czech Republic	49.4167	16.2667	536
	Belmonte-4-94	CS76095	Italy	42.1167	12.4833	218
	Sha / Ara-1	CS76382	Afghanistan	37.29	71.3	4,058
	Sij-2	CS76380	Uzbekistan	41.45	70.05	2,698
	Col-0	CS76778	United States of America	38.3	-92.3	182
	AH-7	-	China	31.19968	115.8067	518
Relict	Tibet-0	CS79062	China	29.68976	91.1861	4,317
	Etna-2	CS76487	Italy	37.69	14.98	1,660
	Meh-0	CS799916	Morocco	33.9561	-4.0515	1,320
	Elk-1	CS799922	Morocco	32.5352	-6.015	1,231
	Ket-10	CS799912	Morocco	34.9608	-4.6661	1,607
	Arb-0	CS799926	Morocco	31.4199	-7.5262	1,097

Supplementary Table 2. Summary of the 32 *Arabidopsis thaliana* ecotypes DNA sequencing data.

Name	Number of HiFi reads	Total data (Gb)	Reads length N50 (bp)	Mean reads length	Sequence coverage[1] (X)
Yilong-0	363,459	6.65	19,035	18,295	48.29
Bor-1	299,373	3.95	15,642	13,177	28.69
Cdm-0	164,271	2.62	16,862	15,934	19.03
Got-22	345,355	4.43	14,685	12,825	32.17
Kondara	341,635	5.10	15,053	14,916	37.04
Kz-9	326,232	4.18	14,288	12,806	30.36
LL-0	352,220	4.90	15,573	13,912	35.58
Mammo-1	173,628	2.33	15,451	13,443	16.92
Ms-0	389,833	5.38	14,790	13,800	39.07
Pra-6	240,797	3.44	14,379	14,271	24.98
Pu2-23	352,556	5.14	14,834	14,582	37.33
Sij-1	296,021	3.89	15,104	13,154	28.25
Sorbo	558,573	4.61	9,787	8,261	33.48
TueSB30-3	499,105	8.22	16,966	16,465	59.69
Hs-0	280,189	3.63	15,007	12,955	26.36
LI-OF-095	317,489	3.81	14,078	12,014	27.67
Per-1	301,006	4.13	15,340	13,714	29.99
St-0	194,285	3.07	16,515	15,805	22.29
Kelsterbach-2	424,029	5.53	14,572	13,039	40.16
Nz-1	400,667	5.50	15,013	13,730	39.94
Dra-2	444,675	6.88	15,653	15,479	49.96
Belmonte-4-94	242,616	3.61	15,003	14,884	26.22
Sha / Ara-1	147,138	2.18	14,961	14,839	15.83
Sij-2	260,428	3.74	14,578	14,379	27.16
Col-0	237,301	4.44	19,185	18,724	32.24
AH-7	291,133	3.82	14,842	13,122	27.74
Tibet-0	349,873	5.99	17,608	17,134	43.50
Etna-2	849,782	6.86	9,382	8,072	49.82
Meh-0	902,680	7.99	10,117	8,850	58.02
Elk-1	857,356	8.15	10,493	9,504	59.19
Ket-10	936,164	8.28	12,176	8,848	60.13
Arb-0	821,175	7.75	10,397	9,431	56.28

[1]Depth was calculated under the estimate of a genome size of 137.70 Mb.

Supplementary Table 3. **Summary of the 11** ***Arabidopsis thaliana*** **ecotypes RNA sequencing data and DNA re-sequencing data for Tibet-0 and Yilong-0.**

**Name**	**Number of clean reads**	**Total data (Gb)**	**Reads length (bp)**	**Insert size (bp)**	**Tissue**	**Library**
Yilong-0	37,664,356	5.65	2x150	380	Leaf	RNA
LI-OF-095	37,104,946	5.57	2x150	380	Leaf	RNA
Kelsterbach-2	35,826,004	5.37	2x150	380	Leaf	RNA
Dra-2	35,761,084	5.36	2x150	380	Leaf	RNA
Belmonte-4-94	34,019,716	5.10	2x150	380	Leaf	RNA
AH-7	33,541,202	5.03	2x150	380	Leaf	RNA
Tibet-0	38,939,972	5.84	2x150	380	Leaf	RNA
Meh-0	36,202,862	5.43	2x150	380	Leaf	RNA
Elk-1	37,121,446	5.57	2x150	380	Leaf	RNA
Ket-10	39,128,482	5.87	2x150	380	Leaf	RNA
Arb-0	40,749,242	6.11	2x150	380	Leaf	RNA
Yilong-0	44,662,924	6.70	2x150	380	Leaf	DNA
Tibet-0	60,291,072	9.04	2x150	380	Leaf	DNA

Supplementary Table 4. Summary of the 21 *Arabidopsis thaliana* ecotypes RNA sequencing data downloaded from NCBI SRA database.

Name	Number of clean reads	Total data (Gb)	Reads length (bp)	SRA number	Tissue
Bor-1	50,350,855	5.04	100	SRX1734500	Leaf
Cdm-0	27,206,548	2.72	100	SRX1735085	Leaf
Got-22	19,665,985	2.01	2x51	ERX1659469	Leaf
Kondara	32,249,429	1.61	50	SRX244065	Leaf
Kz-9	28,682,458	1.43	50	SRX244069	Leaf
LL-0	22,747,781	2.27	100	SRX1734570	Leaf
Mammo-1	30,691,722	3.07	100	SRX1735104	Leaf
Ms-0	23,945,701	2.44	2x51	ERX1659521	Leaf
Pra-6	46,299,849	4.63	100	SRX1735090	Leaf
Pu2-23	27,962,065	1.40	50	SRX244087	Leaf
Sij-1	8,675,693	0.87	100	SRX1734411	Leaf
Sorbo	14,789,077	1.51	2x51	ERX1659578	Leaf
TueSB30-3	25,926,685	2.59	100	SRX1735133	Leaf
Hs-0	27,313,438	1.37	50	SRX244052	Leaf
Per-1	36,800,151	1.84	50	SRX244083	Leaf
St-0	16,576,939	1.69	2x51	ERX1659588	Leaf
Nz-1	26,722,923	2.67	100	SRX1734610	Leaf
Sha / Ara-1	53,209,654	5.32	100	SRX1734418	Leaf
Sij-2	33,211,699	3.32	100	SRX1734412	Leaf
Col-0	74,711,902	21.77	2x146	SRX1735134	Leaf
Etna-2	61,441,623	6.14	100	SRX1734938	Leaf

Supplementary Table 5. K-mer analysis of the Col-0 genome by using K-mer=17.

Name	K-mer	K-mer number	K-mer depth (X)	Genome size (Mb)	Heterozygous ratio (%)	Repeat (%)
Col-0	17	6,471,685,116	47	137.70	0.215	32.58

Supplementary Table 6. Summary of the 32 *Arabidopsis thaliana* ecotypes genome assembly.

Name	Genome length (Mb)	Contig number	Contig N50 (Mb)	Scaffold N50 (Mb)	BUSCO (%)	Repeat rate (%)
Yilong-0	141.8	35	7.35	27.56	99.2	25.99
Bor-1	135.7	25	10.72	27.16	99.2	24.07
Cdm-0	131.2	37	6.94	25.49	99.3	21.52
Got-22	137.9	34	9.06	27.07	99.3	24.40
Kondara	136.4	28	12.32	25.76	99.3	23.96
Kz-9	132.4	39	6.96	26.36	99.3	22.14
LL-0	138.0	27	10.61	27.35	99.2	25.24
Mammo-1	131.2	51	6.72	26.90	99.2	21.68
Ms-0	136.3	32	9.61	26.53	99.3	24.46
Pra-6	136.6	43	8.22	26.10	99.2	23.25
Pu2-23	141.1	27	12.41	27.06	99.3	26.25
Sij-1	134.6	38	9.02	25.39	99.2	23.61
Sorbo	132.7	53	6.27	23.30	99.1	21.87
TueSB30-3	138.4	23	20.30	26.96	99.2	24.64
Hs-0	137.9	38	7.54	26.96	99.2	24.75
LI-OF-095	132.7	41	7.97	24.62	99.2	21.42
Per-1	136.4	31	9.87	26.74	99.2	23.29
St-0	129.4	55	7.82	24.67	99.2	20.34
Kelsterbach-2	135.4	19	13.22	27.38	99.2	24.00
Nz-1	136.5	22	10.38	27.11	99.2	23.47
Dra-2	138.9	17	14.61	27.08	99.3	24.55
Belmonte-4-94	134.9	22	11.95	26.62	99.1	22.51
Sha / Ara-1	135.6	54	6.28	26.43	99.3	23.86
Sij-2	134.7	36	7.84	26.18	99.2	23.60
Col-0	134.4	30	14.27	26.11	99.2	23.00
AH-7	140.3	39	7.52	25.53	99.2	25.42
Tibet-0	137.2	35	13.73	26.23	99.0	24.77
Etna-2	143.0	48	9.93	27.29	99.1	25.43
Meh-0	140.5	56	5.91	26.79	99.2	25.52
Elk-1	139.2	37	8.66	27.12	99.2	25.38
Ket-10	144.9	30	8.08	27.89	99.2	26.44
Arb-0	138.6	20	17.56	27.08	99.1	24.86

Supplementary Table 7. Summary of the 32 *Arabidopsis thaliana* ecotypes gene annotation.

Name	Gene number	BUSCO	Functional annotation number	Annotation rate
Yilong-0	27,683	99.4%	25,916	93.62%
Bor-1	27,801	99.3%	26,087	93.83%
Cdm-0	27,666	99.1%	25,963	93.84%
Got-22	27,835	99.2%	26,066	93.64%
Kondara	27,489	99.3%	25,859	94.07%
Kz-9	27,545	99.2%	25,895	94.01%
LL-0	27,778	99.2%	26,066	93.84%
Mammo-1	27,717	99.0%	25,977	93.72%
Ms-0	27,793	99.2%	26,075	93.82%
Pra-6	27,772	99.3%	26,002	93.63%
Pu2-23	27,962	99.2%	26,183	93.64%
Sij-1	27,521	99.2%	25,822	93.83%
Sorbo	27,646	99.1%	25,913	93.73%
TueSB30-3	27,805	99.1%	26,092	93.84%
Hs-0	27,838	99.4%	26,092	93.73%
LI-OF-095	27,865	99.2%	26,086	93.62%
Per-1	27,793	98.9%	26,019	93.62%
St-0	27,775	99.1%	26,018	93.67%
Kelsterbach-2	27,956	99.3%	26,157	93.56%
Nz-1	27,866	99.5%	26,099	93.66%
Dra-2	27,913	99.3%	26,138	93.64%
Belmonte-4-94	27,716	99.2%	25,953	93.64%
Sha / Ara-1	27,471	99.2%	25,802	93.92%
Sij-2	27,535	99.2%	25,856	93.90%
Col-0	28,735	99.7%	26,625	92.66%
AH-7	27,531	99.3%	25,867	93.96%
Tibet-0	27,239	99.1%	25,650	94.17%
Etna-2	27,812	99.1%	26,096	93.83%
Meh-0	27,661	99.0%	25,935	93.76%
Elk-1	27,686	99.5%	26,018	93.98%
Ket-10	27,607	99.0%	25,951	94.00%
Arb-0	27,711	99.2%	26,009	93.86%

Supplementary Table 8. Our gene annotation of 32 *Arabidopsis thaliana* ecotypes' genome assemblies compare with Araport11 gene annotation.

Name	Mapped gene number[1]	Unmapped gene number	New genes or genes with structure variant number	Final gene number
Yilong-0	23,439	4,215	4,244	27,683
Bor-1	24,136	3,518	3,665	27,801
Cdm-0	23,903	3,751	3,763	27,666
Got-22	24,183	3,471	3,652	27,835
Kondara	23,651	4,003	3,838	27,489
Kz-9	23,716	3,938	3,829	27,545
LL-0	24,043	3,611	3,735	27,778
Mammo-1	23,901	3,753	3,816	27,717
Ms-0	23,851	3,803	3,942	27,793
Pra-6	24,144	3,510	3,628	27,772
Pu2-23	24,390	3,264	3,572	27,962
Sij-1	23,738	3,916	3,783	27,521
Sorbo	23,739	3,915	3,907	27,646
TueSB30-3	24,131	3,523	3,674	27,805
Hs-0	24,157	3,497	3,681	27,838
LI-OF-095	24,038	3,616	3,827	27,865
Per-1	23,829	3,825	3,964	27,793
St-0	24,300	3,354	3,475	27,775
Kelsterbach-2	24,404	3,250	3,552	27,956
Nz-1	24,176	3,478	3,690	27,866
Dra-2	24,107	3,547	3,806	27,913
Belmonte-4-94	24,123	3,531	3,593	27,716
Sha / Ara-1	23,673	3,981	3,798	27,471
Sij-2	23,728	3,926	3,807	27,535
Col-0	27,173	481	1,562	28,735
AH-7	23,297	4,357	4,234	27,531
Tibet-0	22,465	5,189	4,774	27,239
Etna-2	23,246	4,408	4,566	27,812
Meh-0	23,016	4,638	4,645	27,661
Elk-1	23,333	4,321	4,353	27,686
Ket-10	22,901	4,753	4,706	27,607
Arb-0	23,404	4,250	4,307	27,711

[1]Complete genes of >90% identity and coverage with Araport11.

Supplementary Table 9. Results of multiple regression between variable genes and 19 BIOCLIM environmental variables with the Monte Carlo permutation test.

	**PC1**	**PC2**	**R^2**	**Pr(>r)**
BIO1	-0.32383	0.94612	0.0295	0.65355
**BIO2**	**0.1971**	**0.98038**	**0.1883**	**0.0444***
BIO3	-0.8503	0.52629	0.0174	0.76999
BIO4	0.76652	0.64222	0.1037	0.20089
BIO5	0.29219	0.95636	0.1838	0.05263
BIO6	-0.96048	-0.27836	0.0152	0.80755
**BIO7**	**0.54363**	**0.83932**	**0.1982**	**0.04078***
BIO8	-0.86952	-0.49391	0.0572	0.42143
BIO9	0.42596	0.90474	0.0735	0.33393
BIO10	0.22402	0.97459	0.0897	0.2556
BIO11	-0.96278	0.27028	0.0188	0.76403
BIO12	0.00403	-0.99999	0.048	0.48691
BIO13	-0.55576	-0.83134	0.0547	0.42956
BIO14	0.38325	-0.92365	0.046	0.49429
BIO15	-0.84062	0.54162	0.0428	0.52047
BIO16	-0.49465	-0.86909	0.0568	0.41375
BIO17	0.43401	-0.90091	0.0512	0.4502
BIO18	-0.62735	-0.77874	0.1301	0.1284
BIO19	0.97388	-0.22708	0.0635	0.37733

Supplementary Table 10. Summary of pan-TE library constructed by 32 *Arabidopsis thaliana* ecotypes genome assembly.

Type	Number of sequences in pan-TE library
Cent/centromeric_repeat	6
DNA/CACTA	14
DNA/DTA	3
DNA/DTC	7
DNA/DTH	3
DNA/DTM	9
DNA/DTT	1
DNA/Harbinger	3
DNA/Helitron	61
DNA/Mariner	7
DNA/MuDR	58
DNA/hAT	22
DNA/unknown	16
LINE/L1	12
LINE/unknown	7
LTR/Copia	294
LTR/Gypsy	84
LTR/Ty3	66
LTR/unknown	34
MITE/DTA	23
MITE/DTC	1
MITE/DTH	5
MITE/DTM	9
SINE/tRNA	5
SINE/unknown	15
Satellite/Satellite	10
rDNA/spacer	1
repeat/unknown	4
Total	780

Supplementary Table 11. Intact LTR-RTs identified in the 32 *Arabidopsis thaliana* ecotypes genome assembly.

Name	Total intact LTR-RTs searched	Copia	Gypsy	Unknown	Whole genome LAI
Yilong-0	1,498	678	640	180	24.92
Bor-1	1,554	606	708	240	24.52
Cdm-0	1,554	672	684	198	20.47
Got-22	1,476	648	642	186	25.75
Kondara	1,560	762	612	186	21.29
Kz-9	1,842	816	624	402	22.75
LL-0	1,794	702	702	390	13.30
Mammo-1	1,578	546	774	258	5.89
Ms-0	1,734	732	696	306	26.72
Pra-6	1,716	702	744	270	26.59
Pu2-23	1,680	726	684	270	19.20
Sij-1	1,582	780	598	204	20.71
Sorbo	1,528	744	610	174	26.01
TueSB30-3	2,082	702	906	474	24.28
Hs-0	1,536	672	636	228	18.50
LI-OF-095	1,344	552	630	162	19.26
Per-1	1,884	840	702	342	9.08
St-0	1,590	606	708	276	12.02
Kelsterbach-2	1,632	600	804	228	14.55
Nz-1	1,632	636	720	276	20.31
Dra-2	1,632	666	714	252	25.89
Belmonte-4-94	1,650	612	750	288	20.96
Sha / Ara-1	1,720	900	664	156	25.40
Sij-2	1,564	780	592	192	25.59
Wa-1	1,518	648	582	288	8.89
Col-0	1,662	648	582	432	9.11
AH-7	1,426	660	634	132	21.02
Tibet-0	1,656	852	504	300	21.45
Etna-2	1,614	696	642	276	18.40
Meh-0	1,536	798	558	180	19.27
Elk-1	1,878	798	756	324	21.70
Ket-10	1,884	864	798	222	26.91
Arb-0	1,638	804	618	216	20.79

Supplementary Table 12. Summary of the graph pan-genome constructed by 32 *Arabidopsis thaliana* ecotype genomes.

Name	Node number	Node total length (bp)	Edge number
Yilong-0	4,288	2,723,335	8,666
Bor-1	2,906	1,866,149	5,699
Cdm-0	6,234	3,634,406	11,965
Got-22	9,539	4,309,907	17,200
Kondara	929	607,155	1,894
Kz-9	4,078	2,919,425	8,231
LL-0	6,341	3,912,693	12,146
Mammo-1	5,999	3,937,871	11,619
Ms-0	3,667	2,666,982	7,220
Pra-6	3,471	2,620,414	6,837
Pu2-23	11,485	4,581,976	20,241
Sij-1	1,965	1,294,515	4,004
Sorbo	3,541	2,370,470	7,255
TueSB30-3	3,416	2,598,179	6,818
Hs-0	18,527	6,662,639	32,260
LI-OF-095	6,063	3,559,427	10,971
Per-1	5,356	3,764,710	10,750
St-0	30,069	8,897,664	51,070
Kelsterbach-2	8,691	3,857,415	15,397
Nz-1	7,590	4,131,644	13,375
Dra-2	4,850	2,724,617	9,578
Belmonte-4-94	6,401	4,037,183	12,190
Sha / Ara-1	1,425	1,359,281	2,901
Sij-2	29	81,101	56
**Col-0 (reference)**	264,421	134,371,593	264,416
AH-7	3,020	1,626,251	6,294
Tibet-0	8,168	5,353,959	17,402
Etna-2	6,304	4,145,369	13,373
Meh-0	7,764	4,467,368	15,817
Elk-1	7,486	4,173,776	15,049
Ket-10	8,086	5,750,331	16,723
Arb-0	6,059	4,264,136	12,275
Total	468,168	243,271,941	649,692

Supplementary Table 13. Summary of the structural variations (SVs) detected in graph-based pan-genome constructed by 32 *Arabidopsis thaliana* ecotype genomes.

SV type	SV number	SV length (bp)	SV influenced gene number	SV influenced gene percentage (%)	SV with TE inserted	SV with TE inserted percentage (%)
Biallelic/Insertion	3,259	5,543,728	2,746	9.56	1,870	57.4
Biallelic/Deletion	2,701	1,086,966	2,098	7.30	775	28.7
Biallelic/Divergent	26,132	7,508,504	13,694	47.66	3,084	11.80
Multiallelic	29,230	29,647,715	9,084	31.61	8,184	28.00

Supplementary Table 14. Summary of SV classification and TE insertion in the 32 *Arabidopsis thaliana* ecotypes genome assembly.

Name	Ecotype specific SV	Total SV	Ecotype specific SV with TE insertion	Total SV with TE insertion
Yilong-0	311	12,074	99	2,118
Bor-1	411	9,494	166	1,730
Cdm-0	794	10,429	240	1,776
Got-22	404	9,725	134	1,743
Kondara	151	10,762	49	1,895
Kz-9	602	10,862	175	1,939
LL-0	712	10,095	254	1,864
Mammo-1	677	10,139	242	1,789
Ms-0	454	10,062	166	1,794
Pra-6	441	9,438	151	1,785
Pu2-23	424	8,577	137	1,601
Sij-1	6	10,831	5	1,912
Sorbo	294	10,683	99	1,924
TueSB30-3	429	9,640	142	1,763
Hs-0	481	9,196	165	1,626
LI-OF-095	430	9,707	158	1,797
Per-1	570	10,568	176	1,904
St-0	457	8,878	174	1,678
Kelsterbach-2	462	8,828	182	1,604
Nz-1	410	9,416	156	1,702
Dra-2	411	9,458	119	1,688
Belmonte-4-94	557	9,409	208	1,758
Sha / Ara-1	244	10,968	97	1,963
Sij-2	1	10,829	1	1,917
**Col-0 (reference)**	-	-	-	-
AH-7	753	12,820	167	2,081
Tibet-0	3,109	16,281	685	2,424
Etna-2	2,239	14,461	550	2,251
Meh-0	1,803	14,935	465	2,242
Elk-1	1,165	12,672	327	2,043
Ket-10	2,521	15,963	594	2,392
Arb-0	1,316	12,711	381	2,014

Supplementary Table 15. Summary of the structural variations (SVs) location detected in graph-based pan-genome constructed by 32 *Arabidopsis thaliana* ecotype genomes.

SV type	Gene Upstream 2k	Gene Downstream 2k	CDS	Intron	Total
Biallelic/Insertion	2,210	1,891	276	304	4,423
Biallelic/Deletion	1,496	1,438	446	473	3,313
Biallelic/Divergent	10,668	10,024	2,271	5,542	17,531
Multiallelic	7,684	6,529	1,583	2,193	12,445
Total	16,367	15,032	4,164	7,242	22,407

Supplementary Table 16. Primers used for RT-qPCR.

Primer name	Sequence (5’ to 3’)
EIF4A-QPCR-F	CGTGACCCGTGATGATGAGA
EIF4A-QPCR-R	AGTACGGCAGAGCAAACACA
CCR1-QPCR-F	GGAACCGTACGGAATCCAGATGATC
CCR1-QPCR-R	CCTCGTAGTCCTGAAGATCTGCTTTG
KNAT3-QPCR-F	ATGGCGTTTCATCACAATCATCTCTCAC
KNAT3-QPCR-R	CTTCTTGGAAATGTTGTTGCTGTTGC
WH1-QPCR-F	TGGTTCCAATCTCAAAGAGATTCTC
WH1-QPCR-R	GTCTTGAGAAACAAGGATTCCCAGG
HPCA1-QPCR-F	AACAGTAACTCGGCCCGTTT
HPCA1-QPCR-R	AGATCCGCAACTCGGACAAG

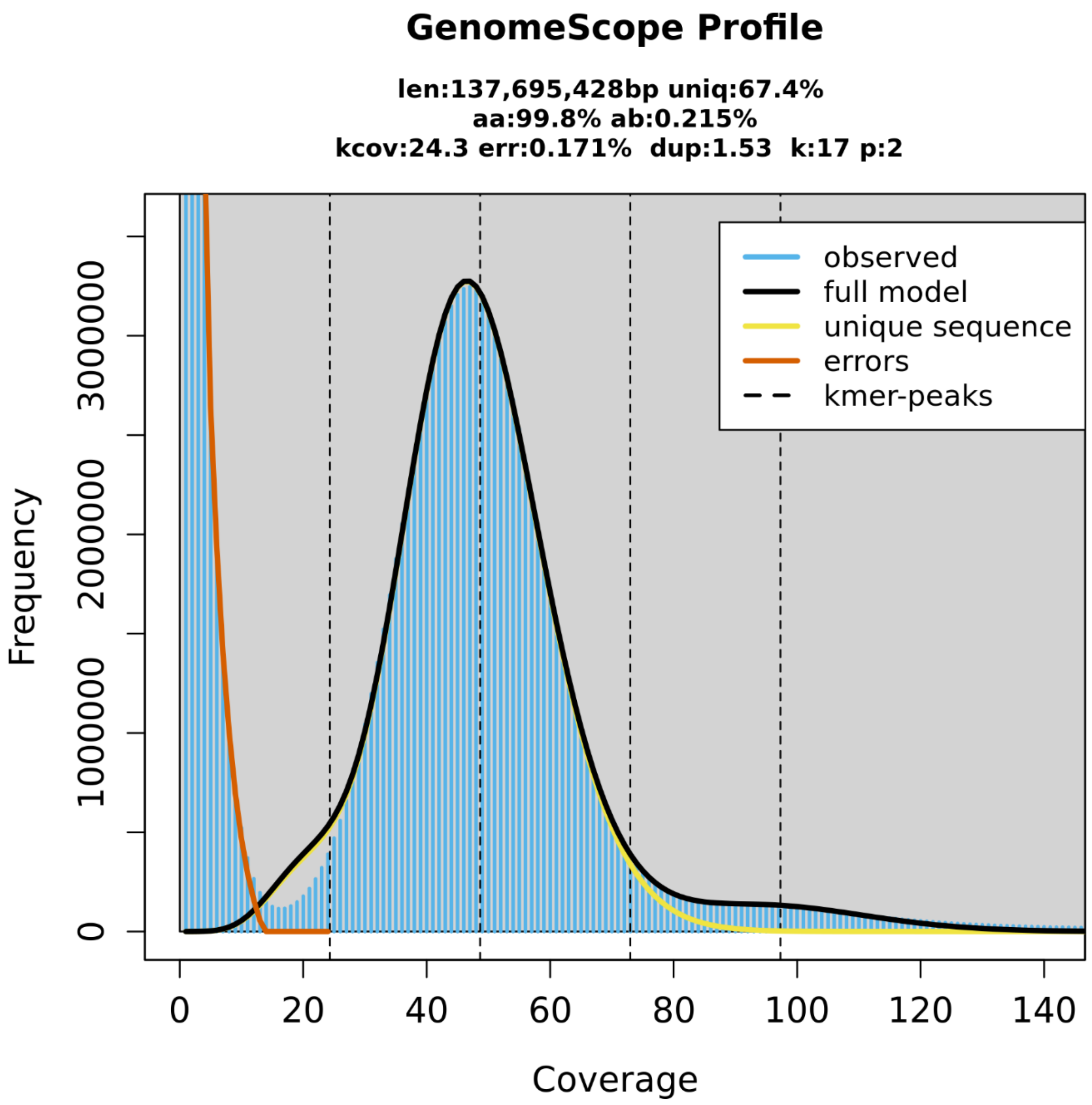

Supplementary Fig. 1. Estimation of Col-0 genome size by K-mer analysis. The figure shows the frequency of 17 k-mers, which are 17 bp sequences from clean reads of short-insert-size libraries. We identified 6,471,685,116 K-mers and the peak of K-mer depth is 47. Genome size can be estimated as (total K-mer number) / (the volume peak). The genome size of Col-0 was thus estimated as 137.70 Mb.

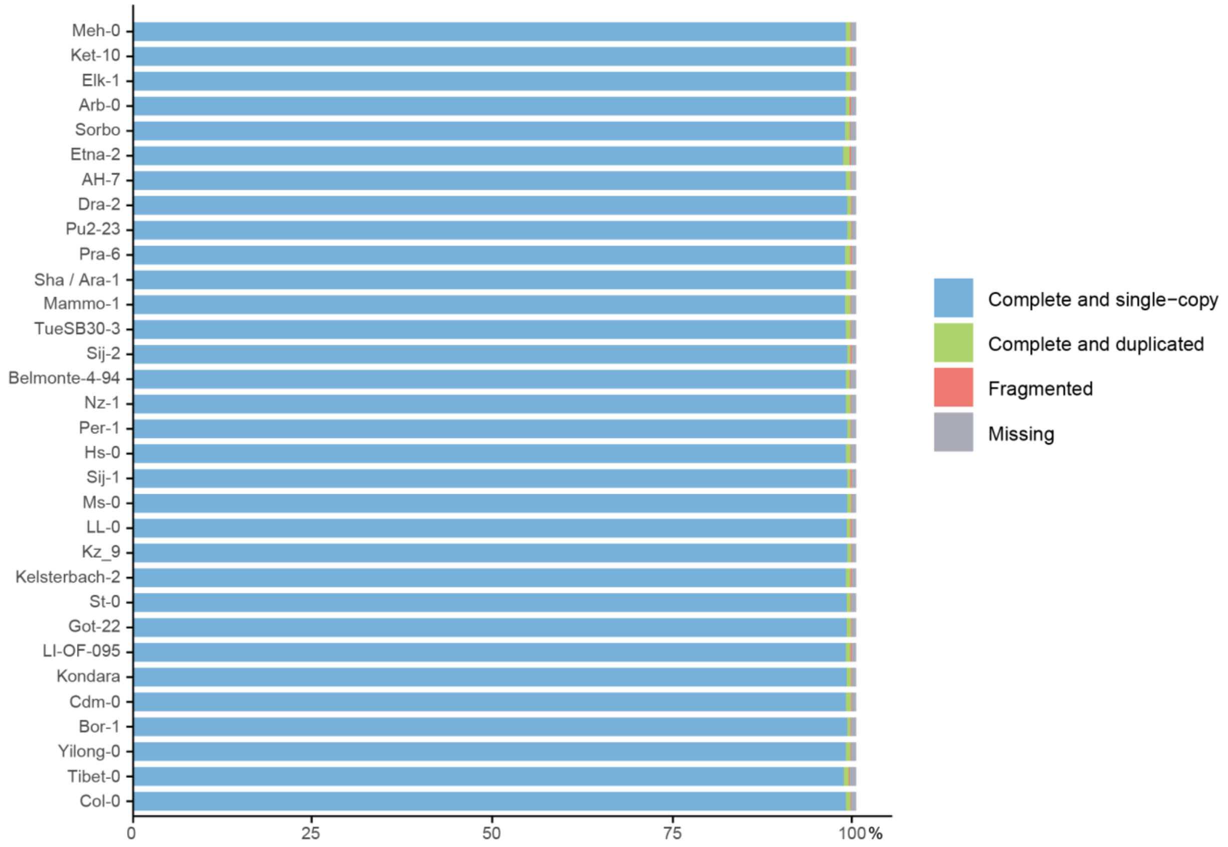

Supplementary Fig. 2. BUSCO assessment of the 32 *Arabidopsis thaliana* ecotypes genome assemblies.

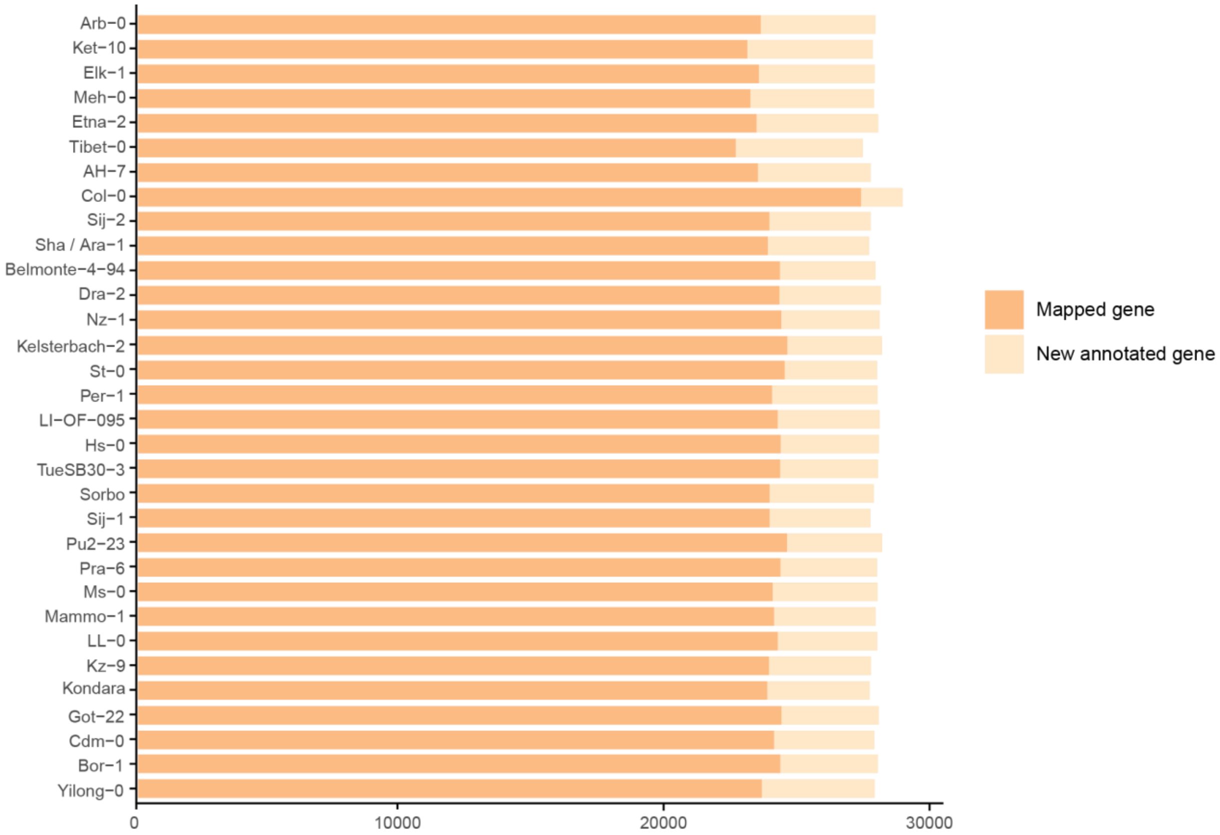

Supplementary Fig. 3. Summary of our 32 assemblies gene annotation compared with Araport11 version gene annotation of Col-0.

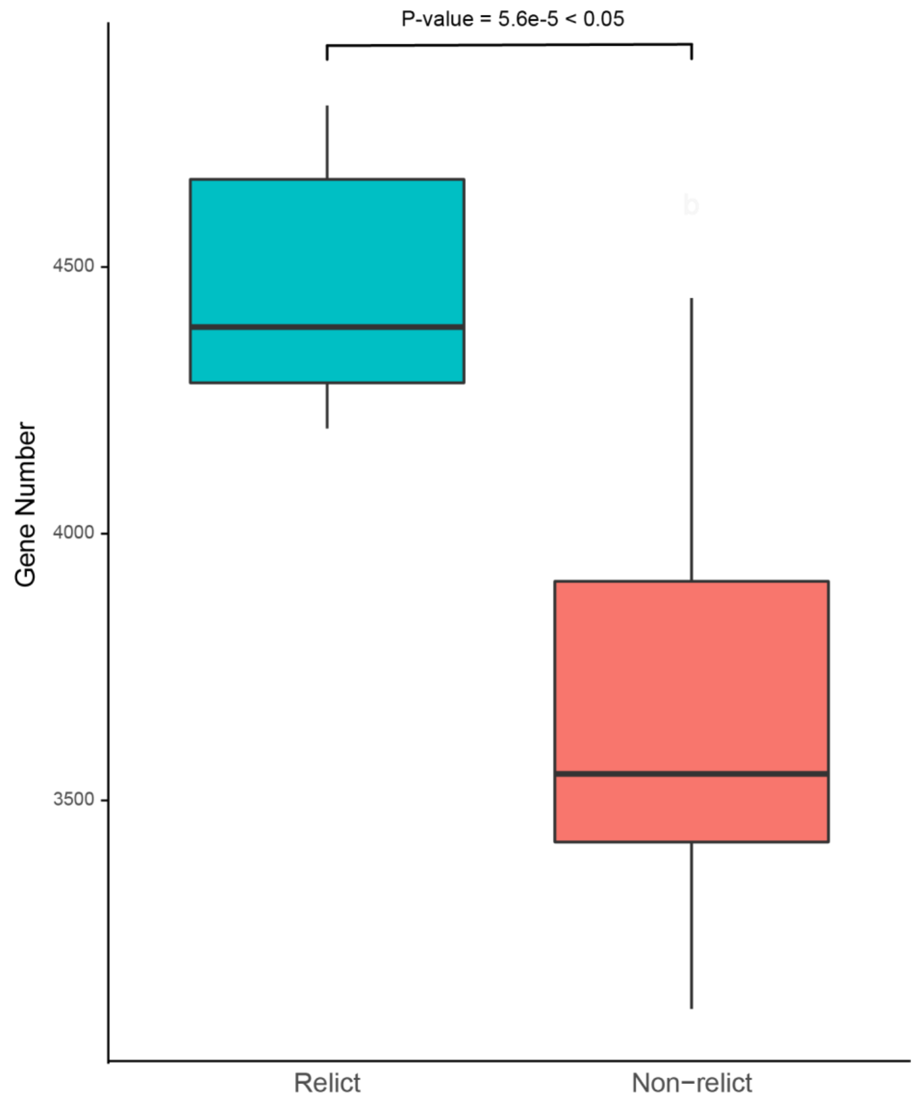

Supplementary Fig. 4. Comparison of genes different from Araport11 between relict ecotypes and non-relict ecotypes. Significance tested by two tailed Wilcoxon method with $p = 5.6e-5 < 0.05$. The middle line of the boxplot is the median, the lower and upper hinges correspond to the first and third quartiles, the upper whisker extends from the hinge to the largest value no further than 1.5 × IQR from the hinge (where IQR is the inter-quartile range) and the lower whisker extends from the hinge to the smallest value at most 1.5 × IQR of the hinge, the outliers are removed. Source data are provided as a Source Data file.

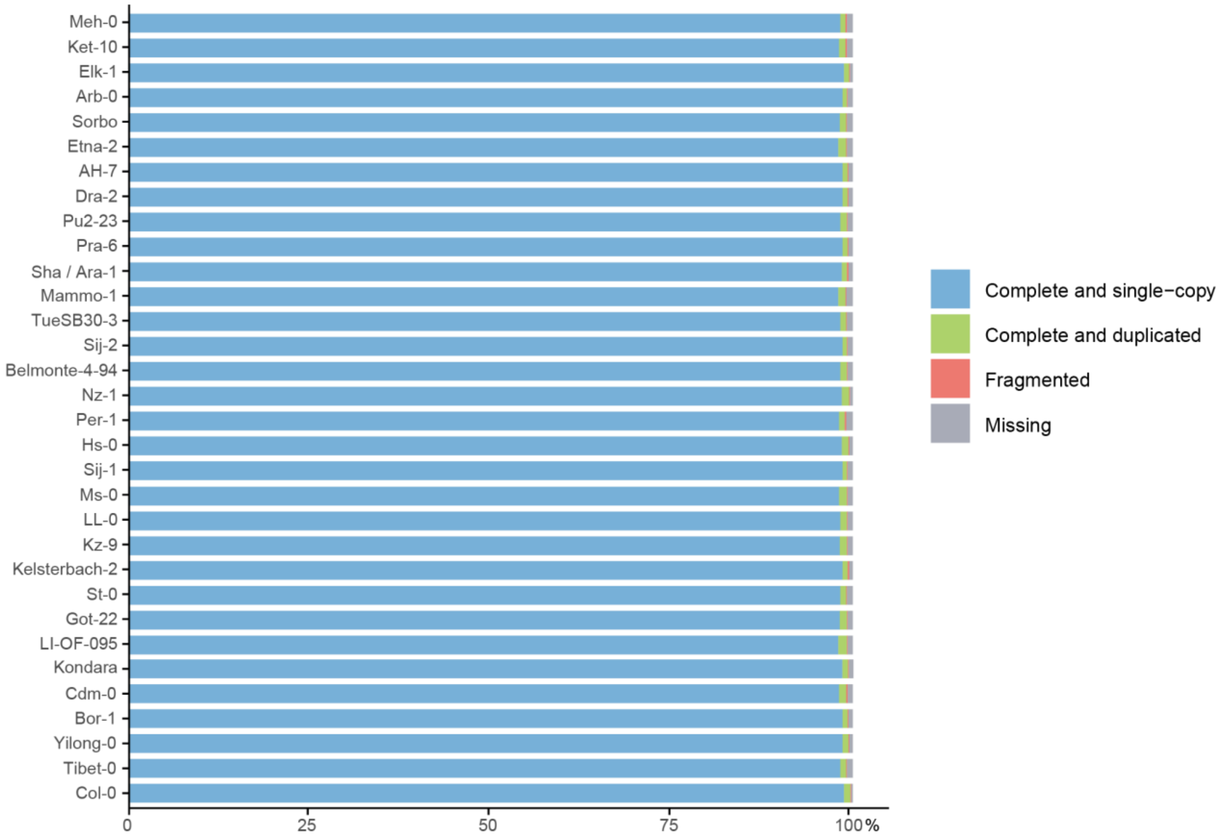

Supplementary Fig. 5. BUSCO assessment of the 32 *Arabidopsis thaliana* ecotypes genome assemblies' gene annotations.

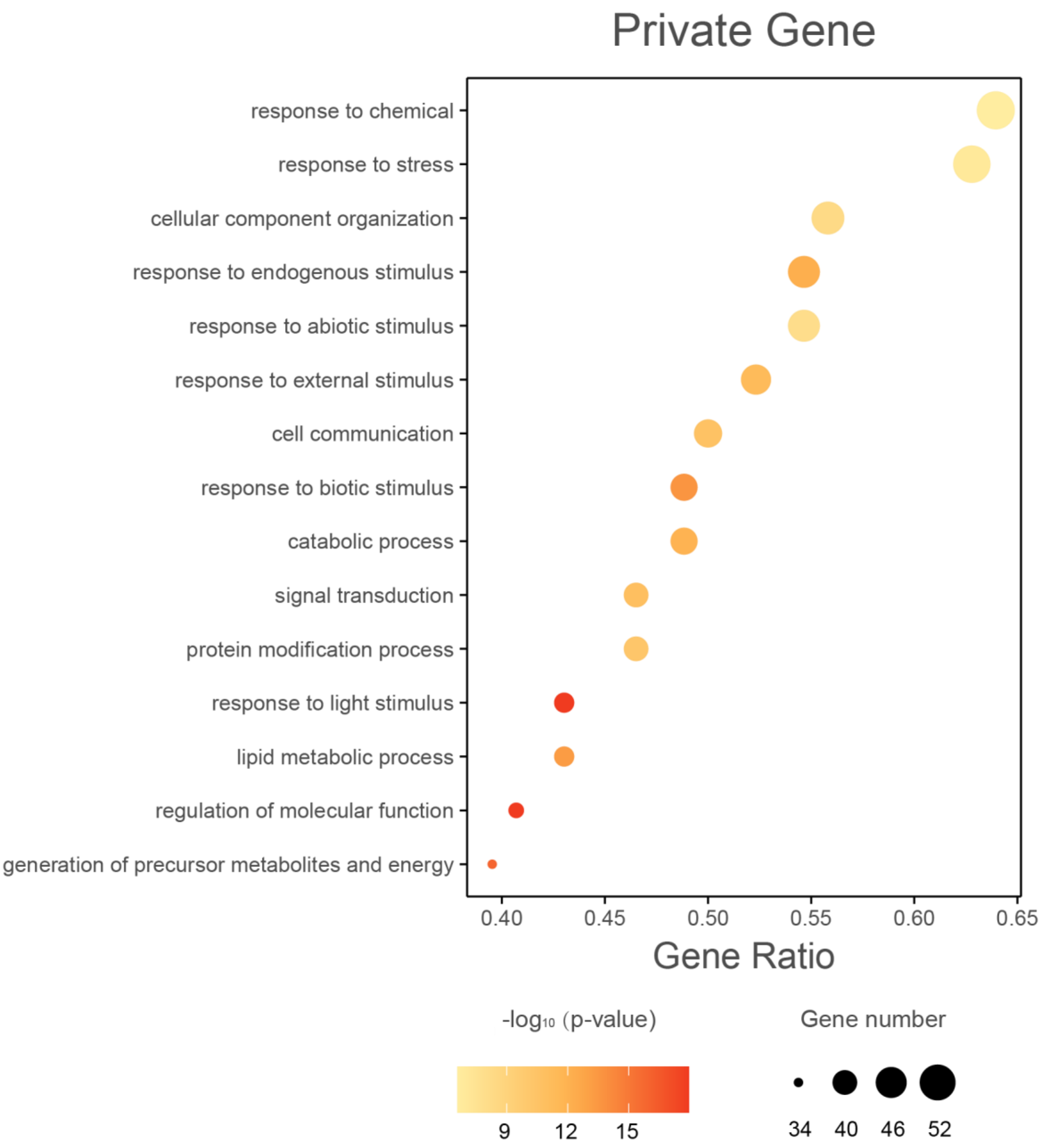

Supplementary Fig. 6. Bubble chart of GO enrichment analysis for private genes. Source data are provided as a Source Data file.

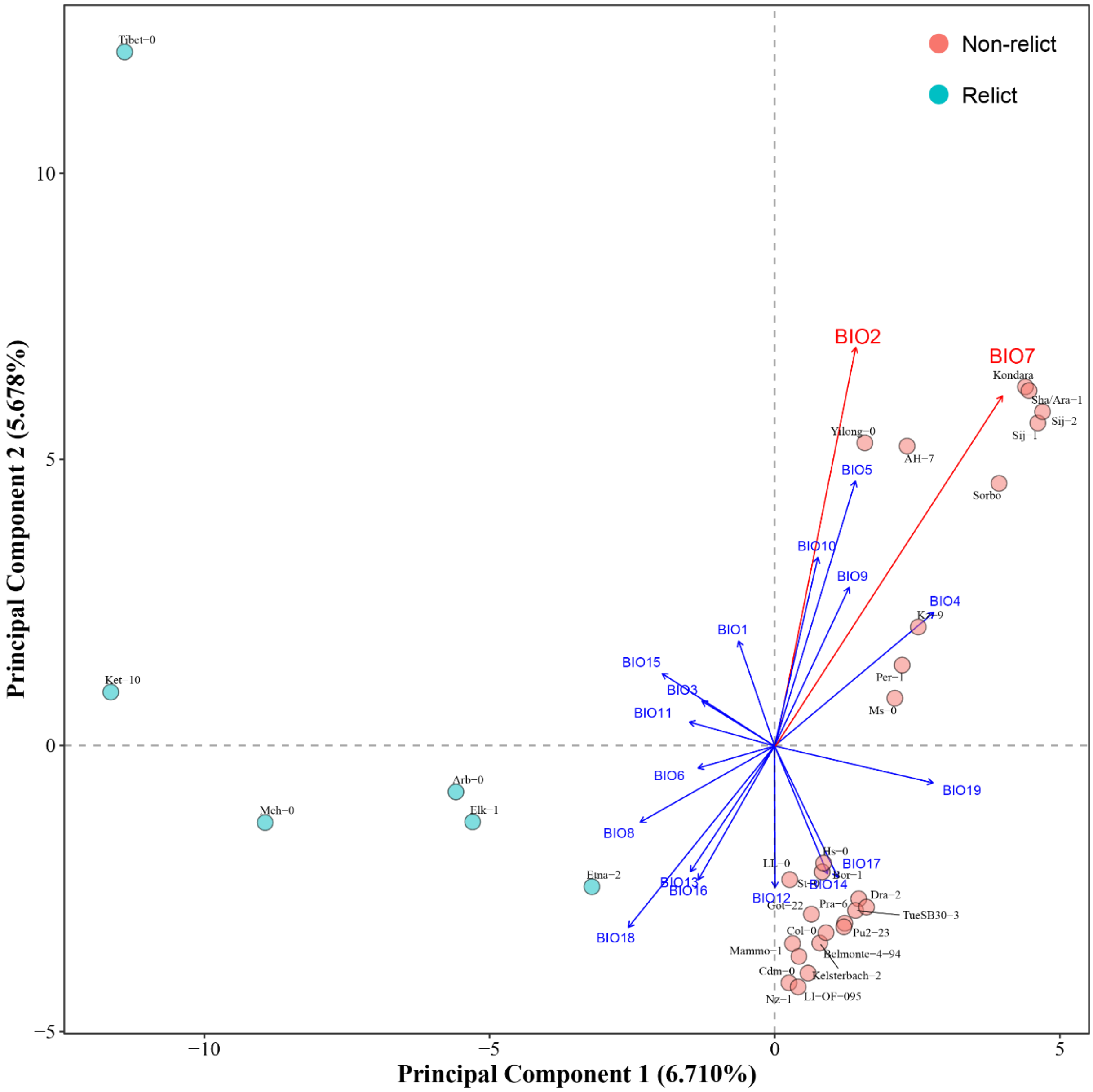

Supplementary Fig. 7. Principal component analysis (PCA) of 32 *Arabidopsis thaliana* ecotypes based on variable gene families and multiple regression of 19 BIOCLIM environmental variables on selected ordination axes. Blue circles displays relict ecotypes, while red circles displays non-relict ecotypes. Arrow in red shows significant assosiated environmental variables. Significance of the regression's coefficient of determination (r2) for each environmental variables was tested by 99,999 times Monte Carlo permutation test. Source data are provided as a Source Data file.

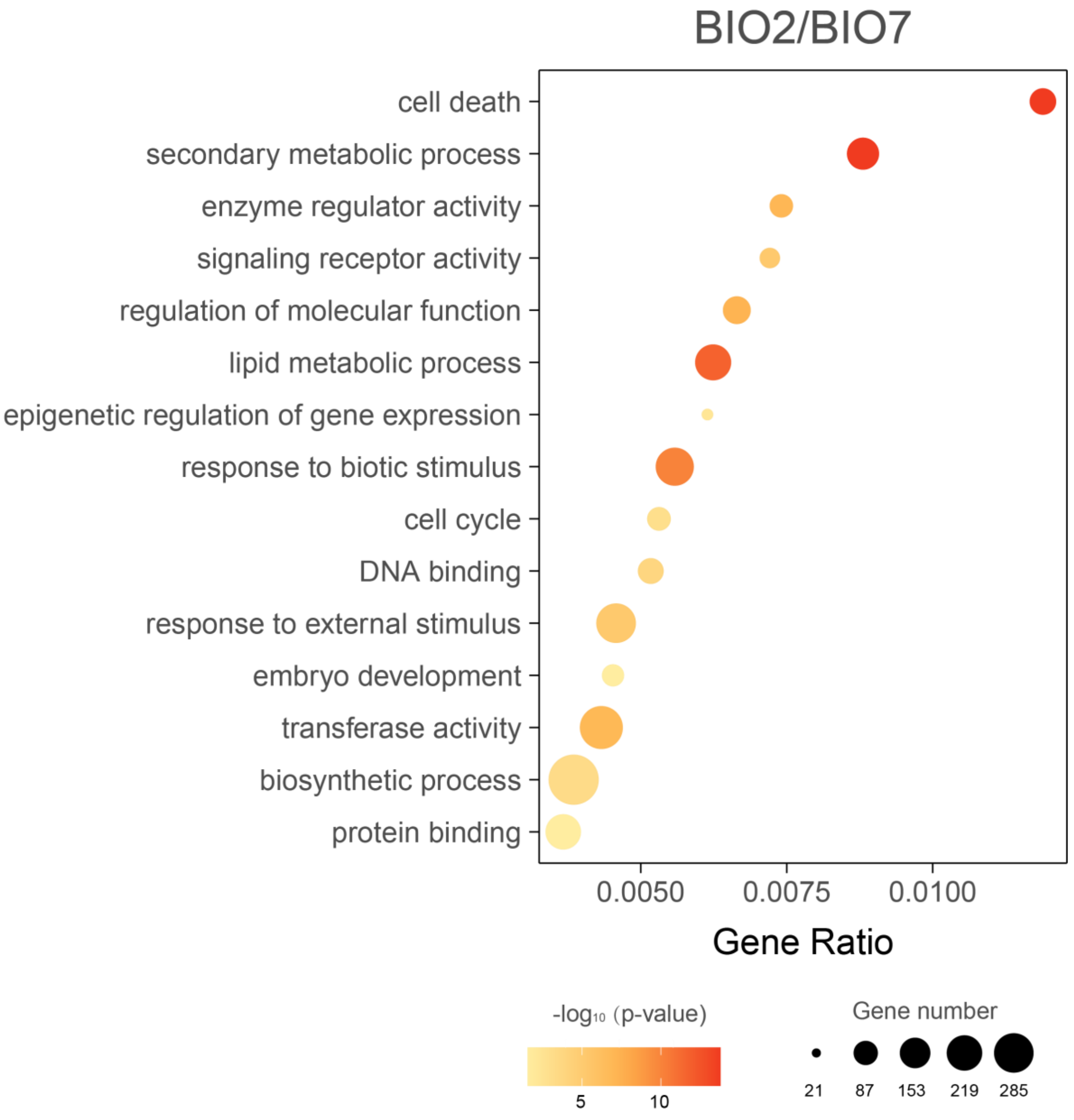

Supplementary Fig. 8. Bubble chart of GO enrichment analysis for 215 variable genes families significantly associated with BIO2 and BIO7. Source data are provided as a Source Data file.

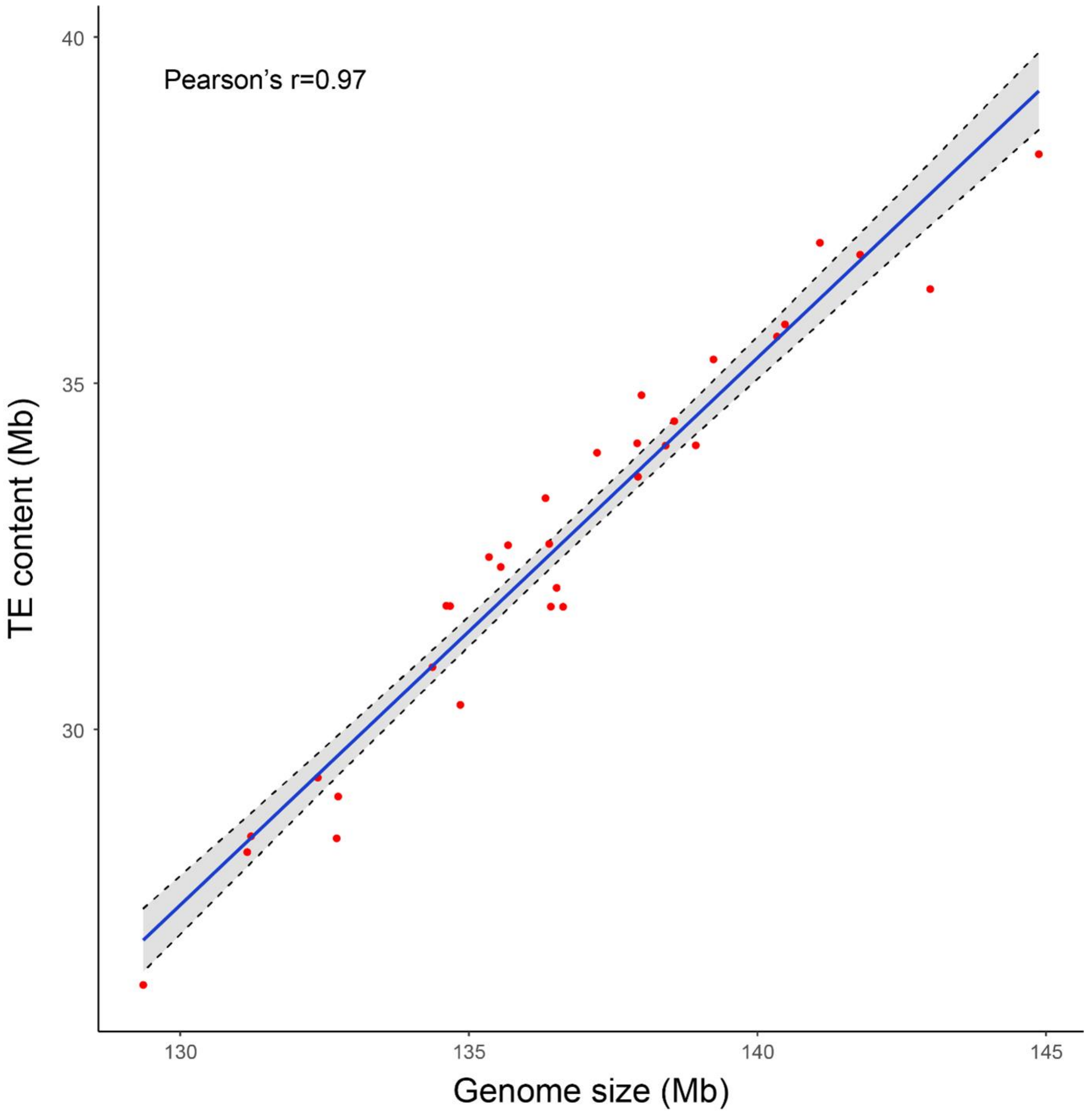

Supplementary Fig. 9. Pearson's correlation coefficient between TE content and genome size. Source data are provided as a Source Data file.

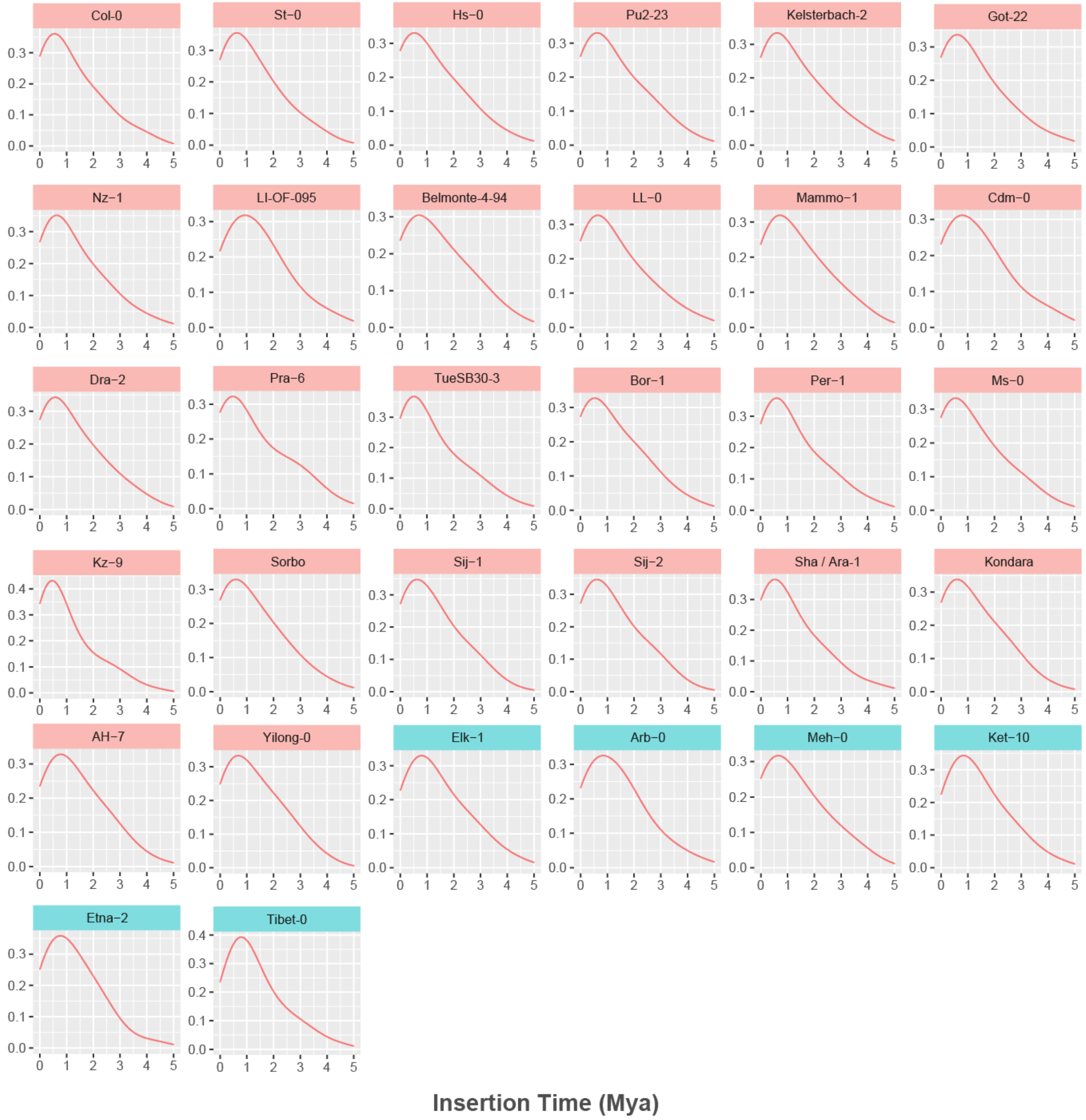

Supplementary Fig. 10. Intact LTR insertion time distribution of each accessions. The x-axis represents the insertion time from million years ago (Mya), y-axis represents the density of the insertion time. Blue rectangle displays relict ecotypes, while red rectangle displays non-relict ecotypes. The mutation rate used for time calculation was $7x10^{-9}$. Source data are provided as a Source Data file.

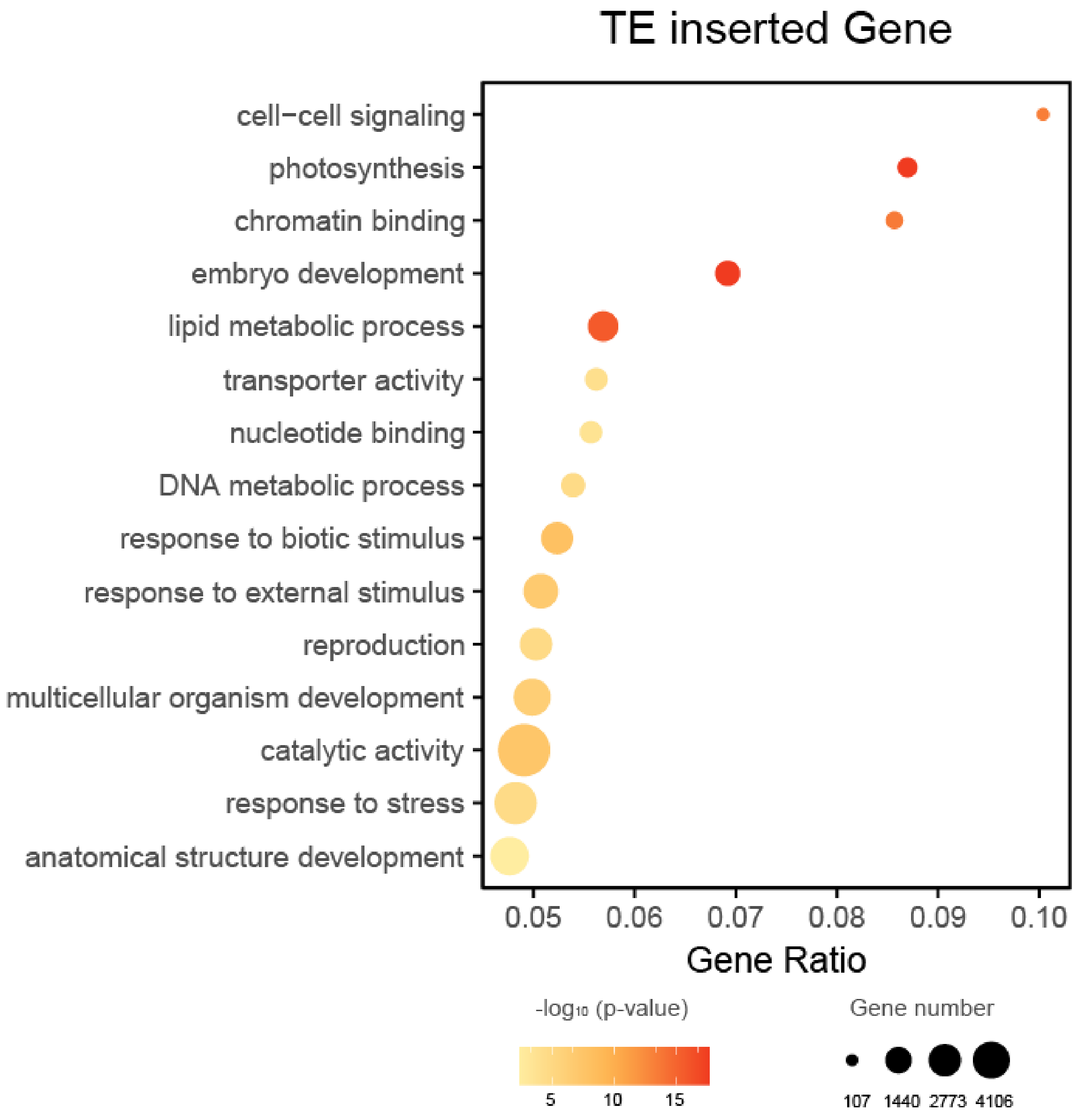

Supplementary Fig. 11. Bubble chart of GO enrichment analysis for TE inserted genes. Source data are provided as a Source Data file.

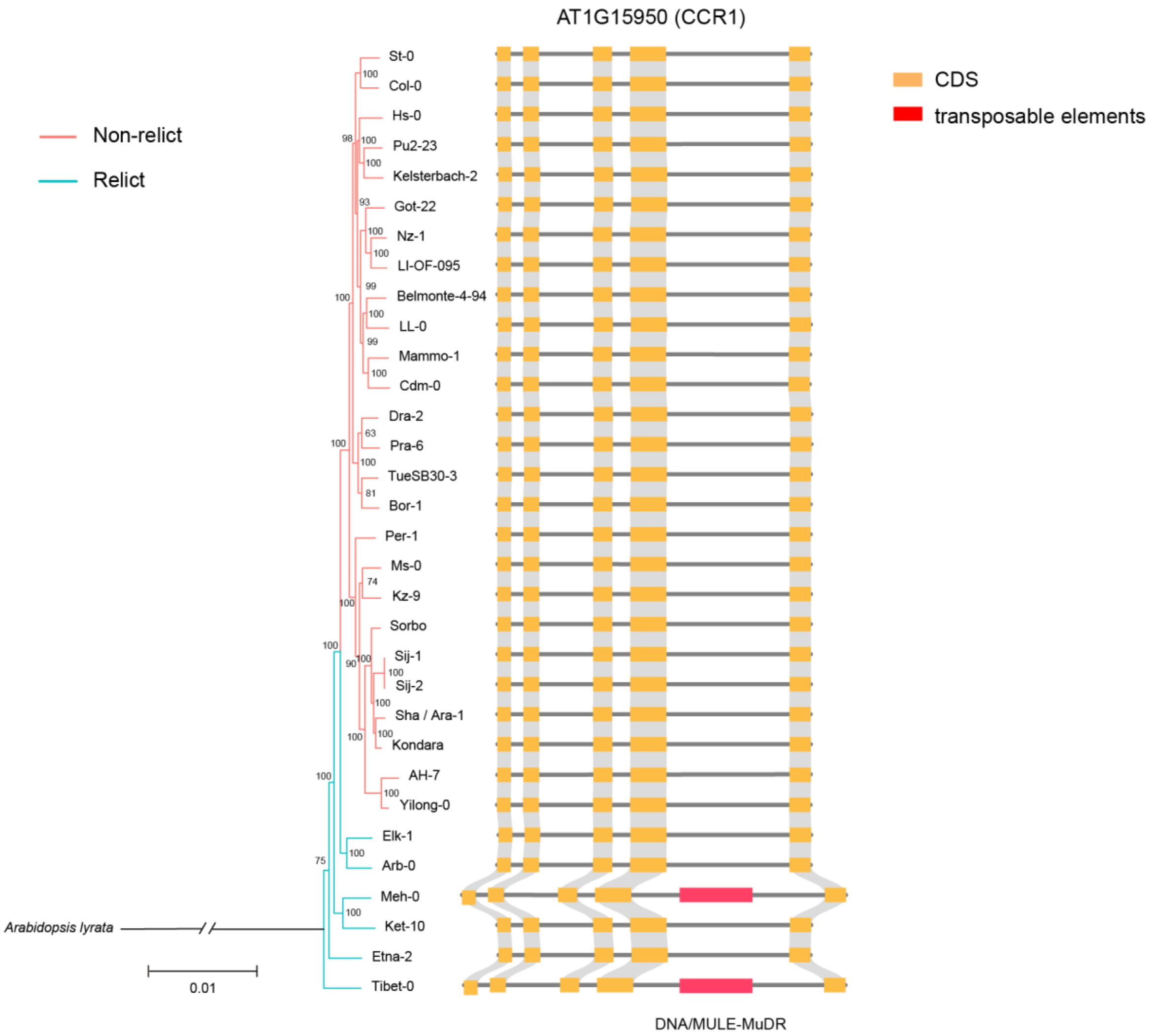

Supplementary Fig. 12. ***AT1G15950 (CCR1)*** **gene structure in** ***Arabidopsis thaliana*** **ecotypes genomes.** Only Tibet-0 and Meh-0 have a DNA transposon insertion in the fourth intron region.

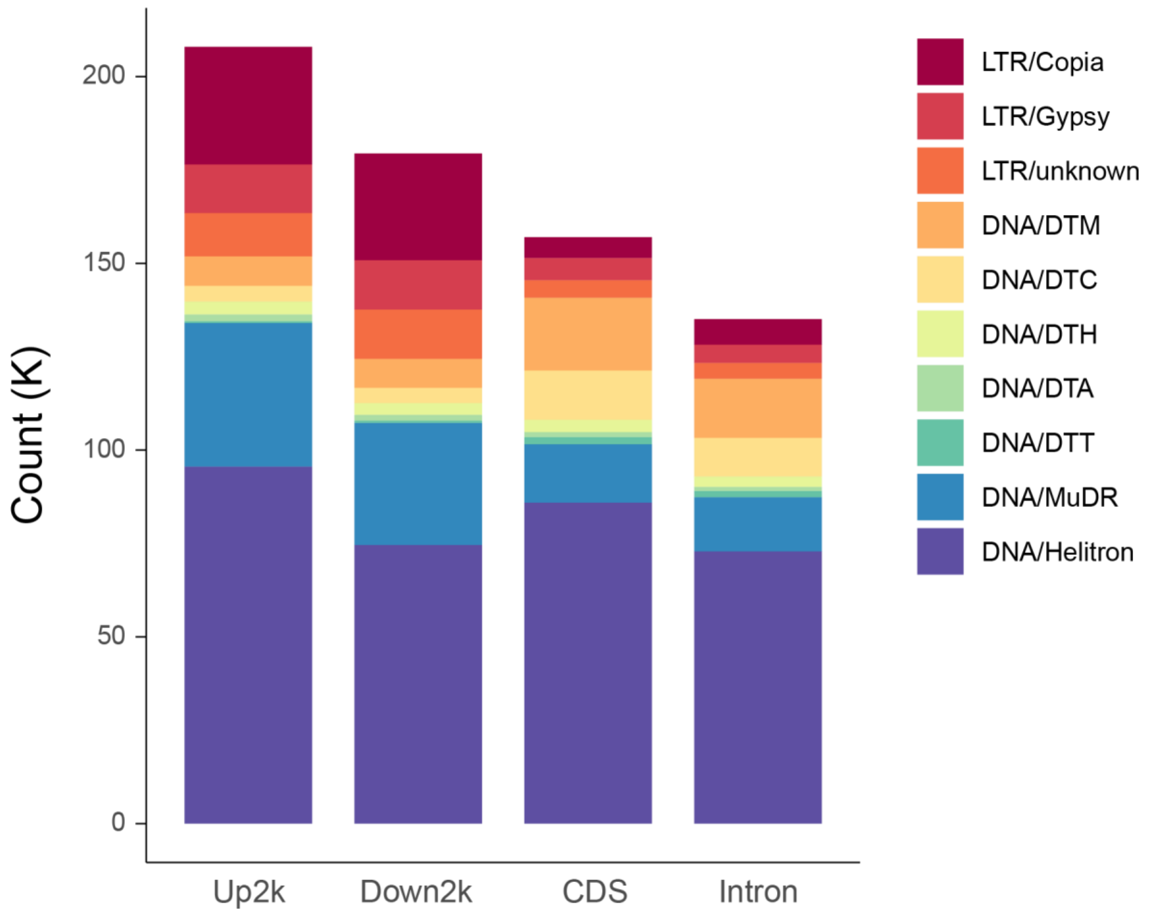

Supplementary Fig. 13. Composition of different TE types inserted around genes.

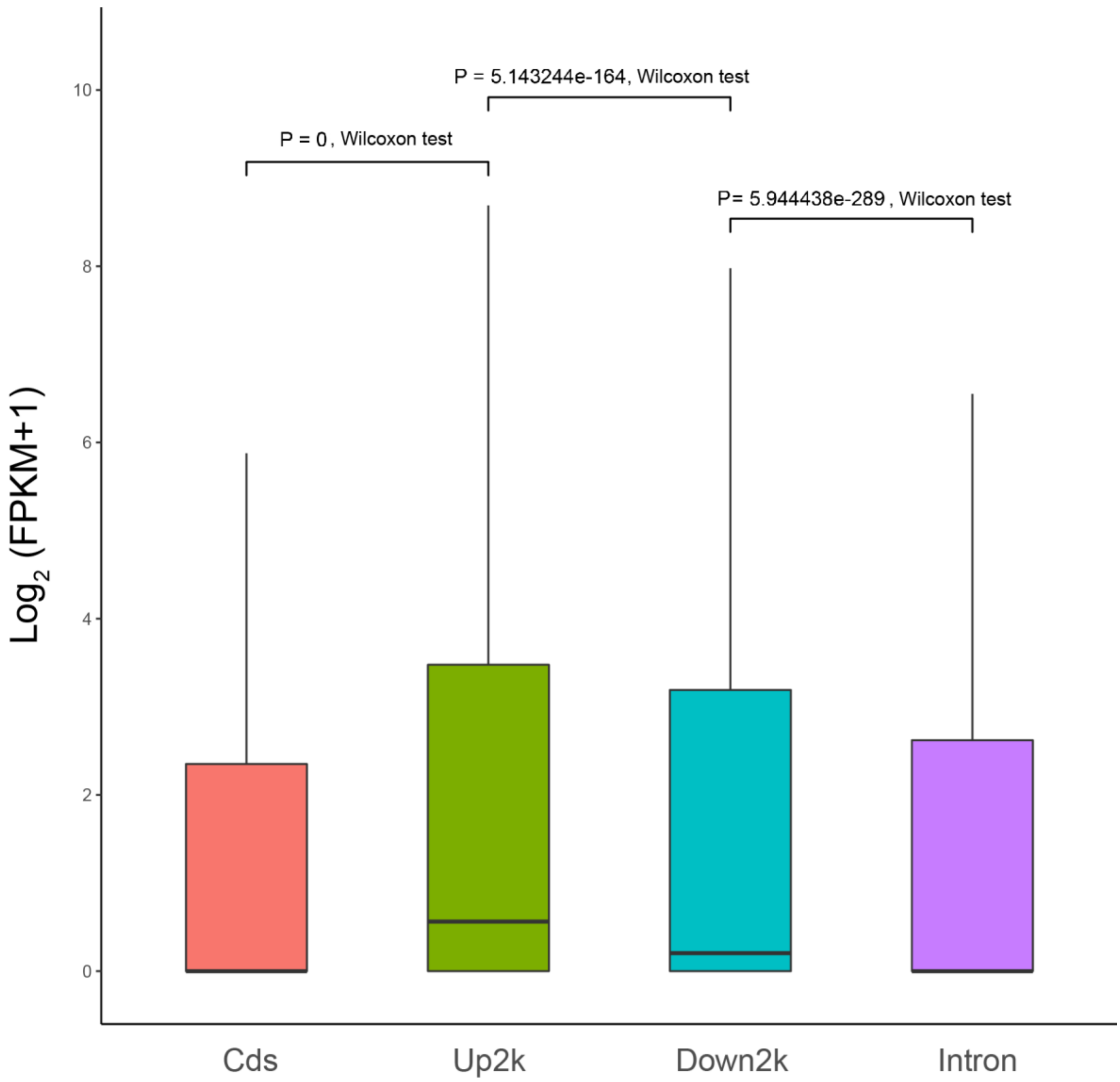

Supplementary Fig. 14. The expression level of genes with TE inserted in different regions. The middle line of the boxplot is the median, the lower and upper hinges correspond to the first and third quartiles, the upper whisker extends from the hinge to the largest value no further than 1.5 × IQR from the hinge (where IQR is the inter-quartile range) and the lower whisker extends from the hinge to the smallest value at most 1.5 × IQR of the hinge, the outliers are removed. Significance tested by two tailed Wilcoxon method (*p* = 0, 5.143244e-164 and 5.944438e-289).

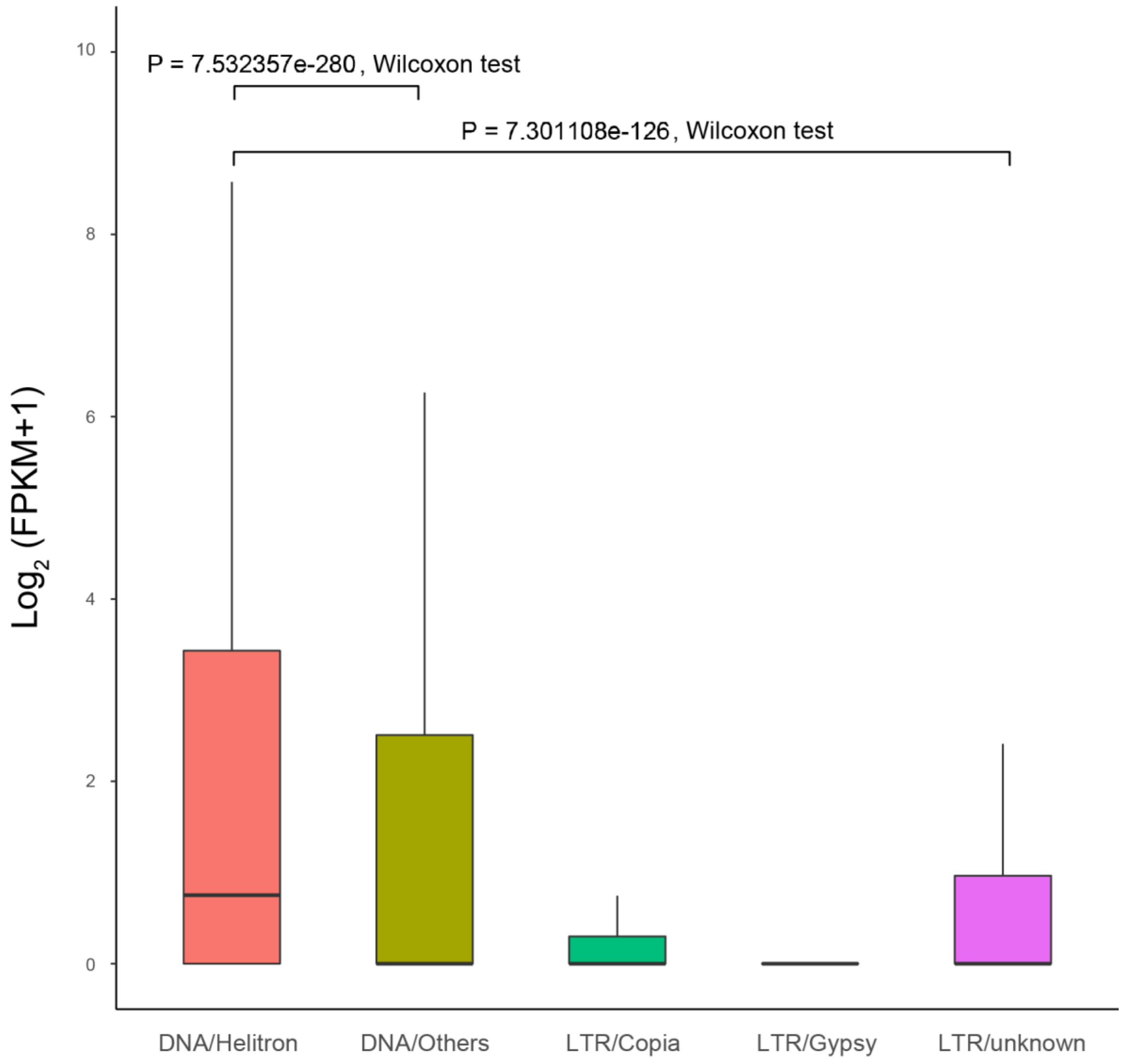

Supplementary Fig. 15. The expression level of genes with different TE types' insertion. The middle line of the boxplot is the median, the lower and upper hinges correspond to the first and third quartiles, the upper whisker extends from the hinge to the largest value no further than 1.5 × IQR from the hinge (where IQR is the inter-quartile range) and the lower whisker extends from the hinge to the smallest value at most 1.5 × IQR of the hinge, the outliers are removed. Significance two tailed tested by Wilcoxon method (p = 7.532357e-280 and 7.301108e-126).

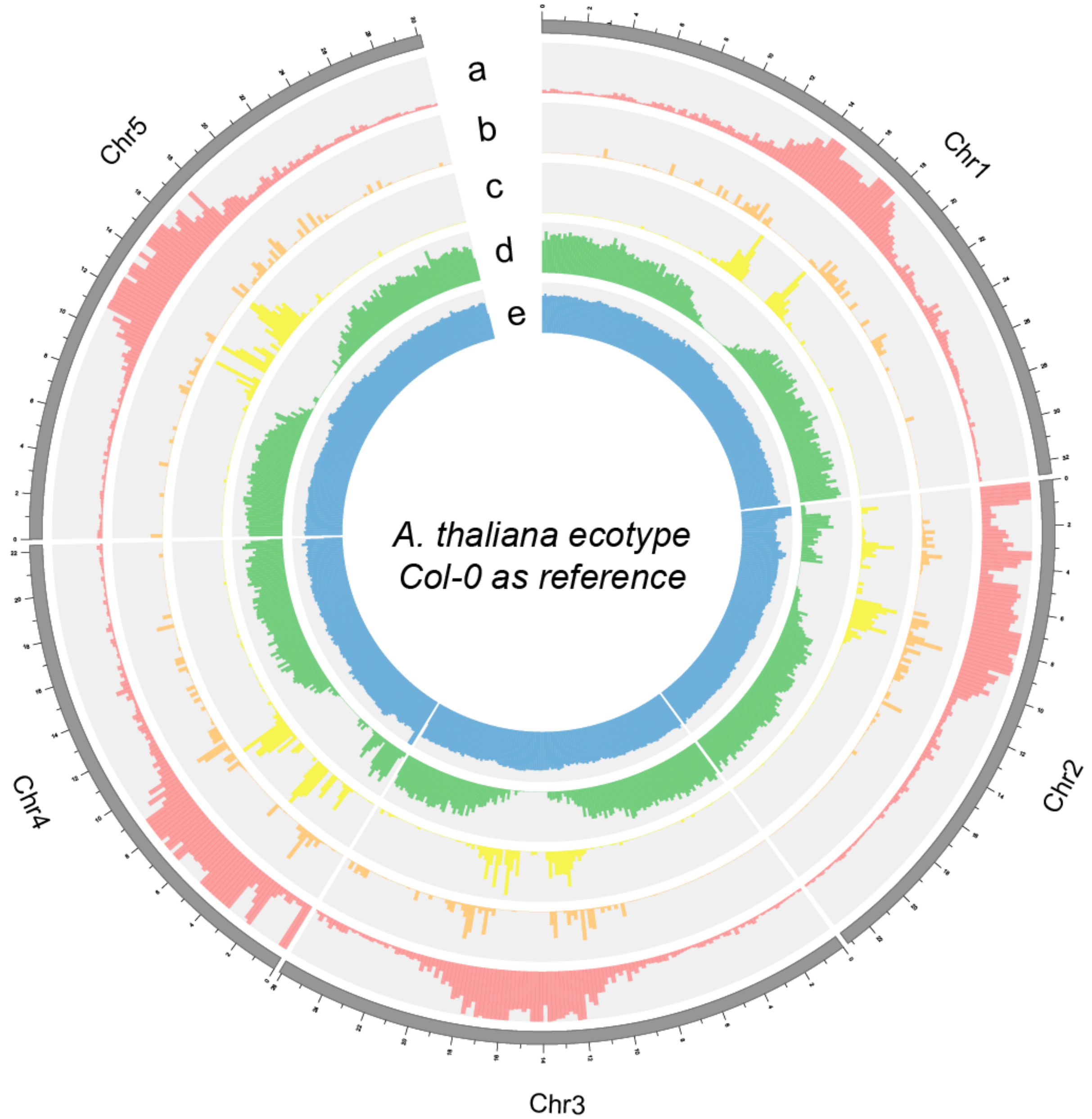

Supplementary Fig. 16. Circos plot of the reference genome Col-0. a. all TE distribution across the whole genome; b. LTR/Copia distribution across the whole genome; c. LTR/Gypsy distribution across the whole genome; d. gene density of the Col-0 genome; e. GC density of the Col-0 genome.

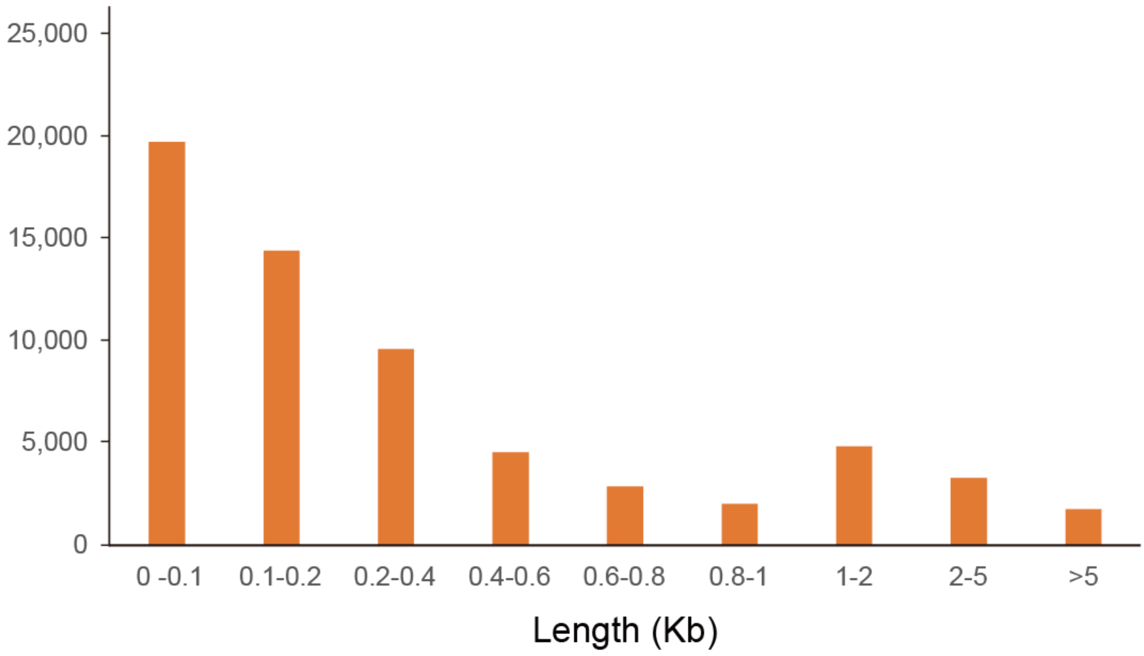

Supplementary Fig. 17. Distribution of the SVs length in graph-based pan-genome constructed by 32 *Arabidopsis thaliana* ecotype genomes.

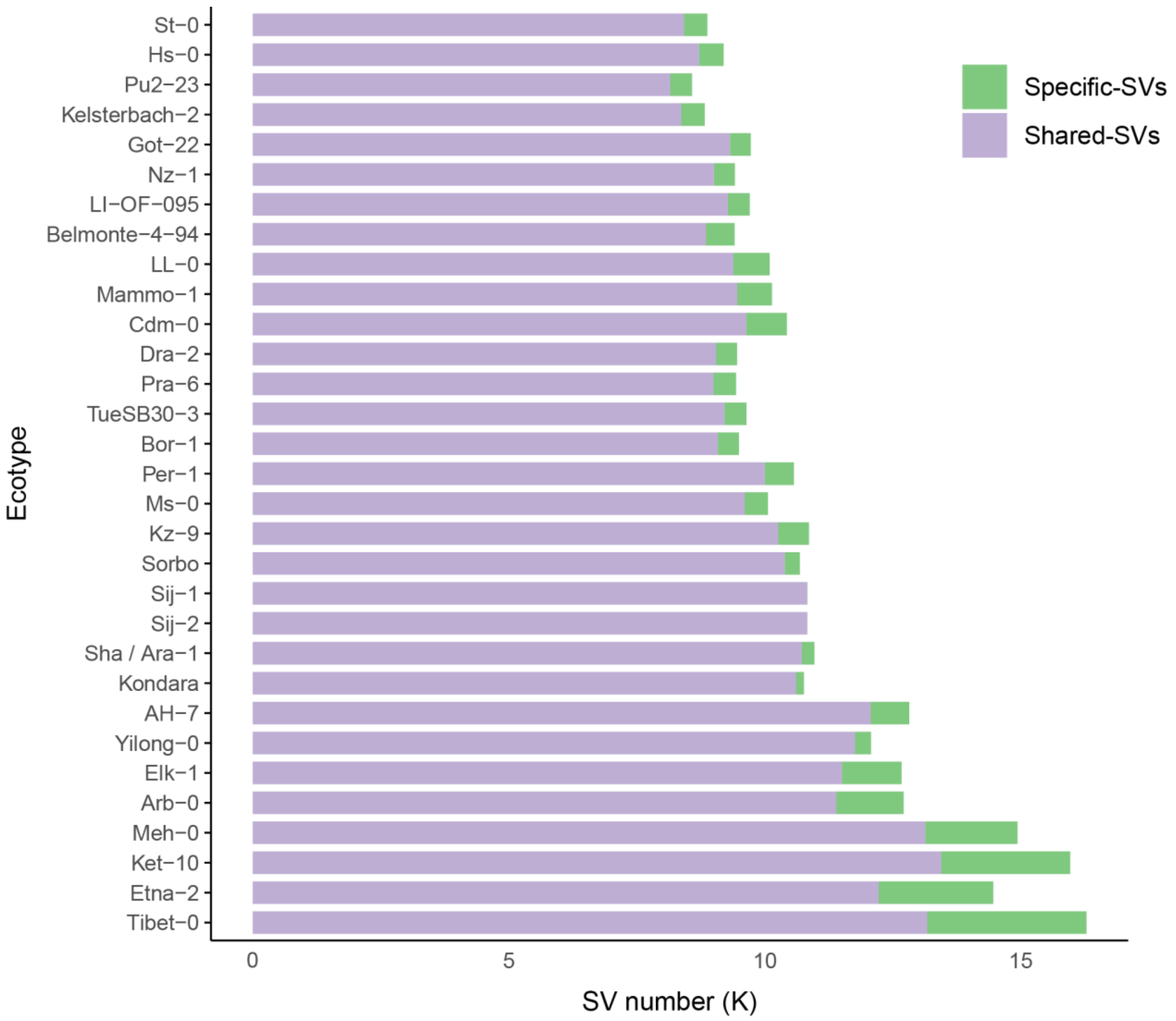

Supplementary Fig. 18. Shared-SVs and ecotype-specific SVs in 32 *A. thaliana* genomes. Blue rectangle displays relict ecotypes, while red rectangle displays non-relict ecotypes.

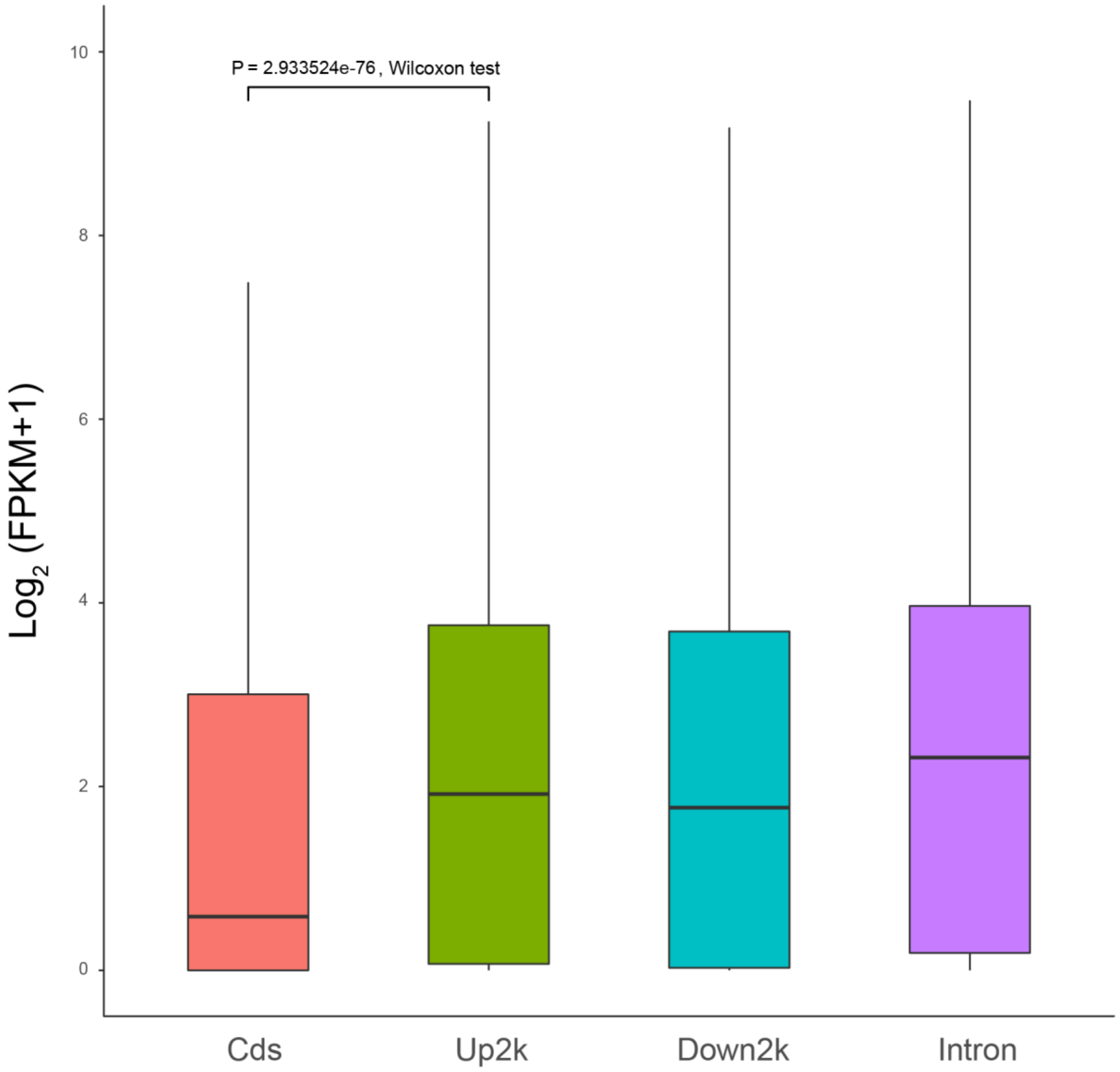

Supplementary Fig. 19. The expression level of genes with SV overlapped in different regions. The middle line of the boxplot is the median, the lower and upper hinges correspond to the first and third quartiles, the upper whisker extends from the hinge to the largest value no further than 1.5 × IQR from the hinge (where IQR is the inter-quartile range) and the lower whisker extends from the hinge to the smallest value at most 1.5 × IQR of the hinge, the outliers are removed. Significance tested by two tailed Wilcoxon method (p = 2.933524e-76).

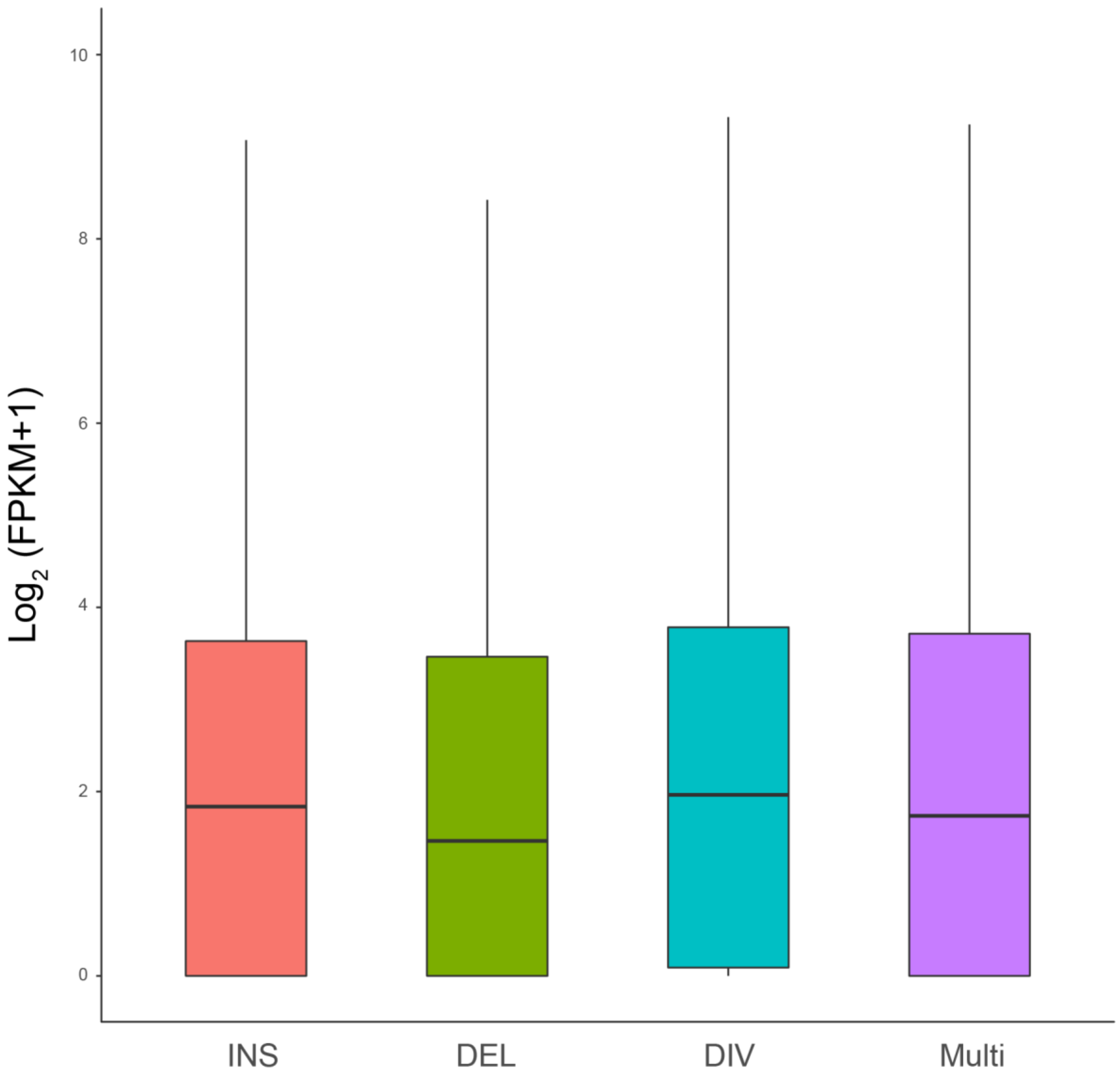

Supplementary Fig. 20. The expression level of genes overlapped with different SV types. The middle line of the boxplot is the median, the lower and upper hinges correspond to the first and third quartiles, the upper whisker extends from the hinge to the largest value no further than 1.5 × IQR from the hinge (where IQR is the inter-quartile range) and the lower whisker extends from the hinge to the smallest value at most 1.5 × IQR of the hinge, the outliers are removed. Significance tested by two tailed Wilcoxon method.

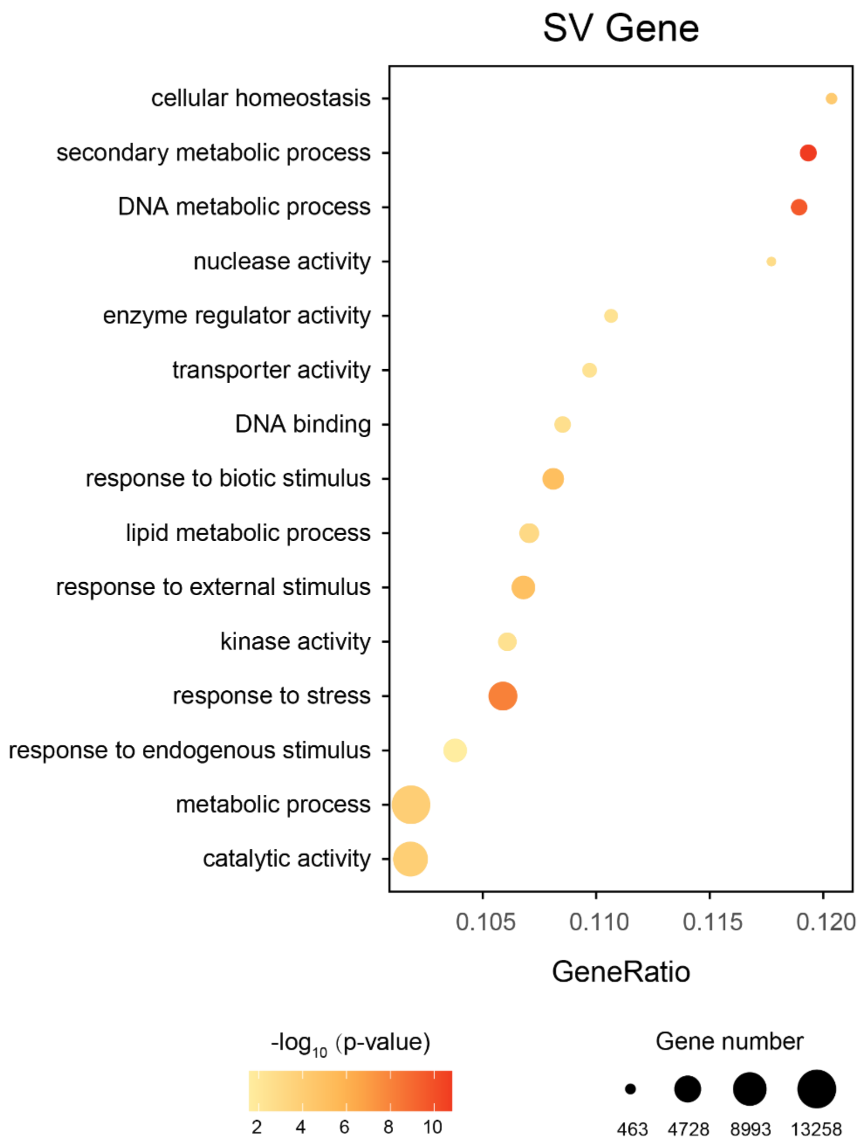

Supplementary Fig. 21. Bubble chart of GO enrichment analysis for SV overlapped genes.

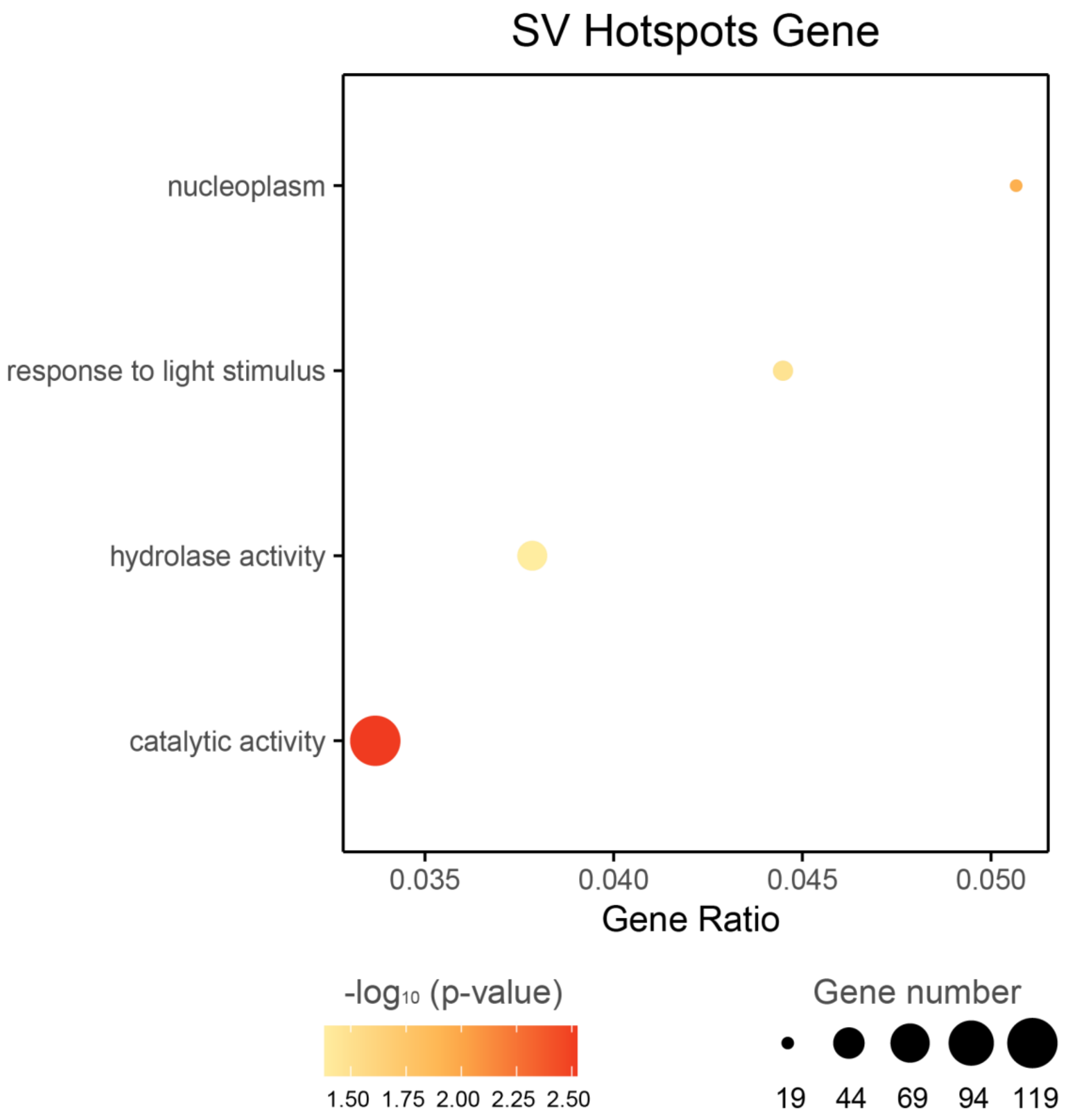

Supplementary Fig. 22. Bubble chart of GO enrichment analysis for genes in SV hotspot region.

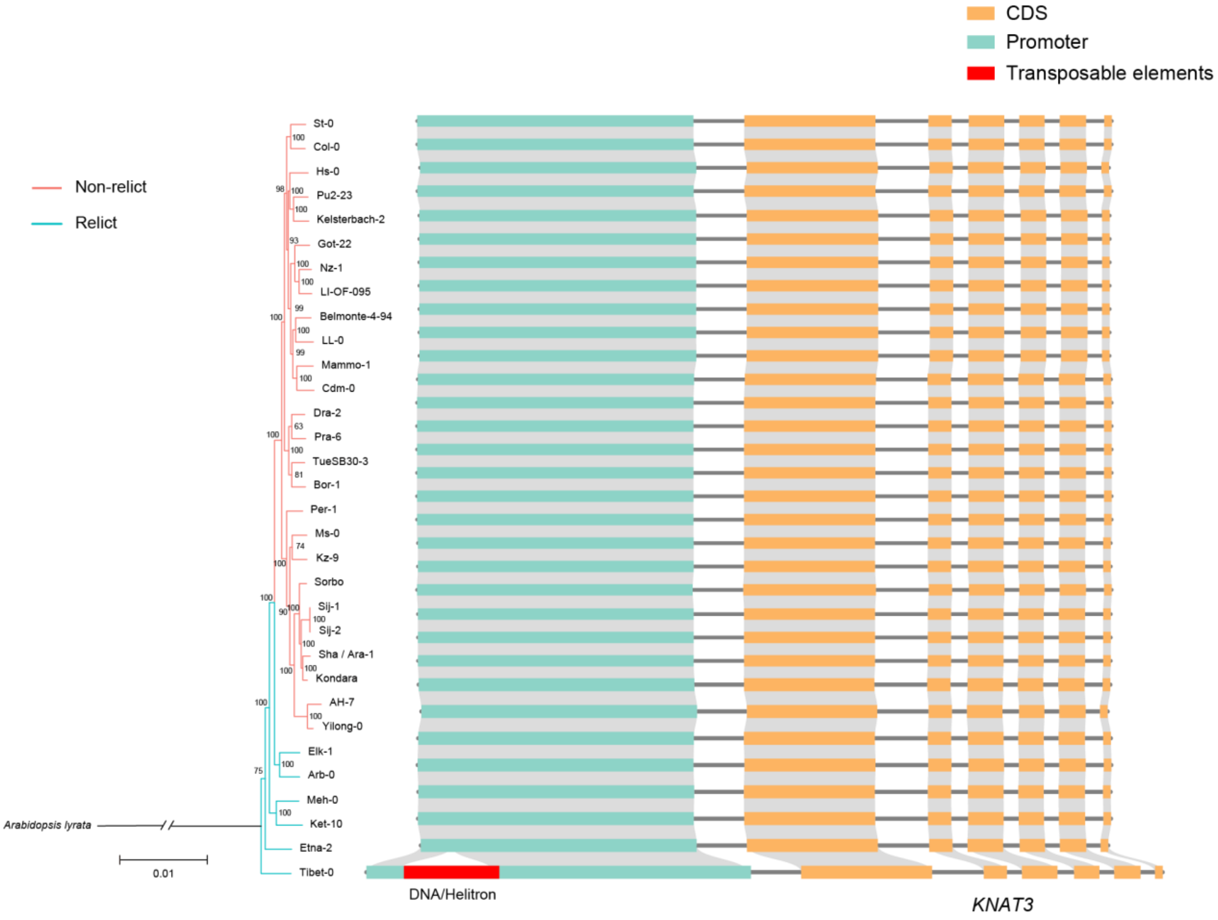

Supplementary Fig. 23. ***AT5G25220 (KNAT3)*** **gene structure in** ***Arabidopsis thaliana*** **ecotypes genomes.** Only Tibet-0 have a DNA transposon insertion in the promoter region.

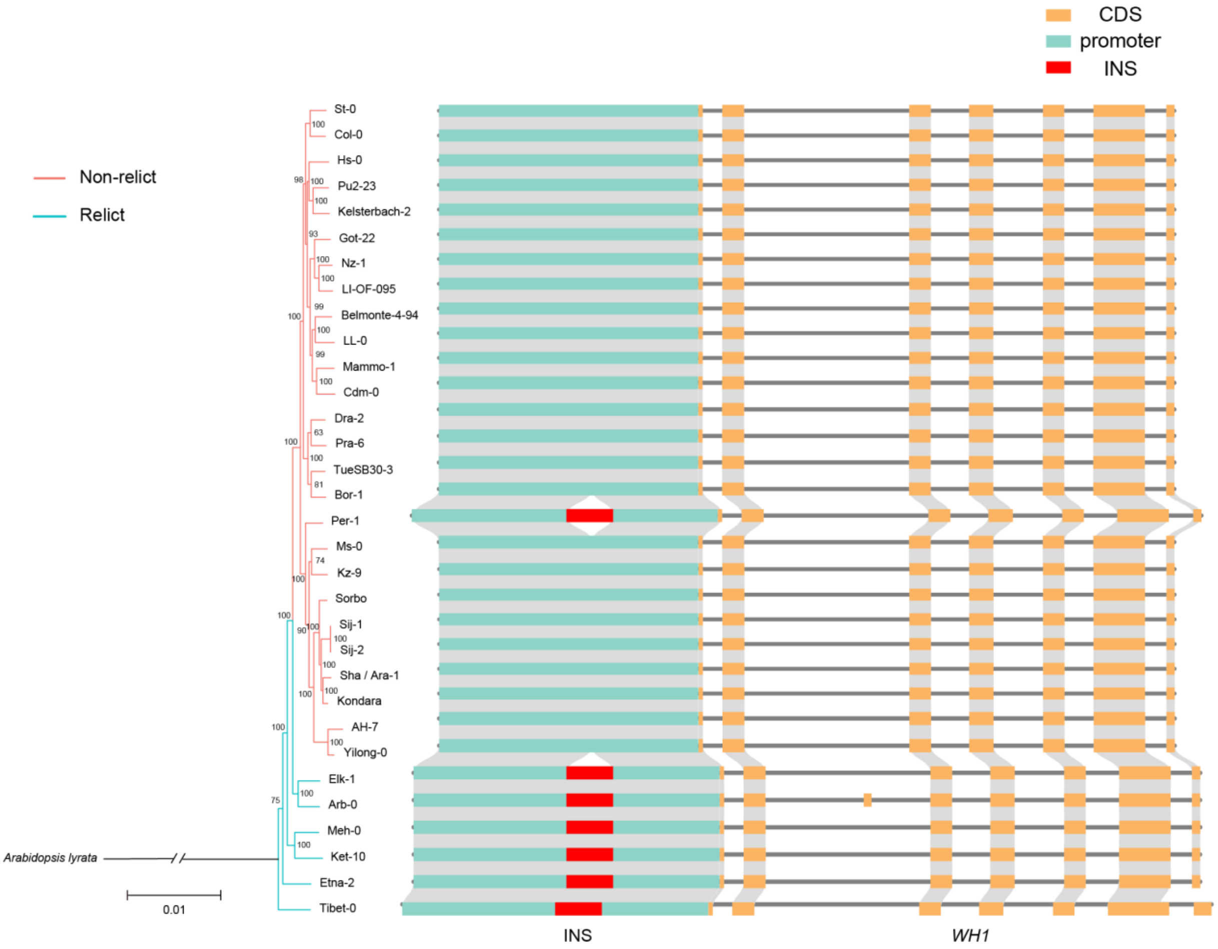

Supplementary Fig. 24. ***AT1G54260 (WH1)*** **gene structure in** ***Arabidopsis thaliana*** **ecotypes genomes.** Only 6 relict ecotypes and Per-1 have a 180 bp insertion in the promoter region.

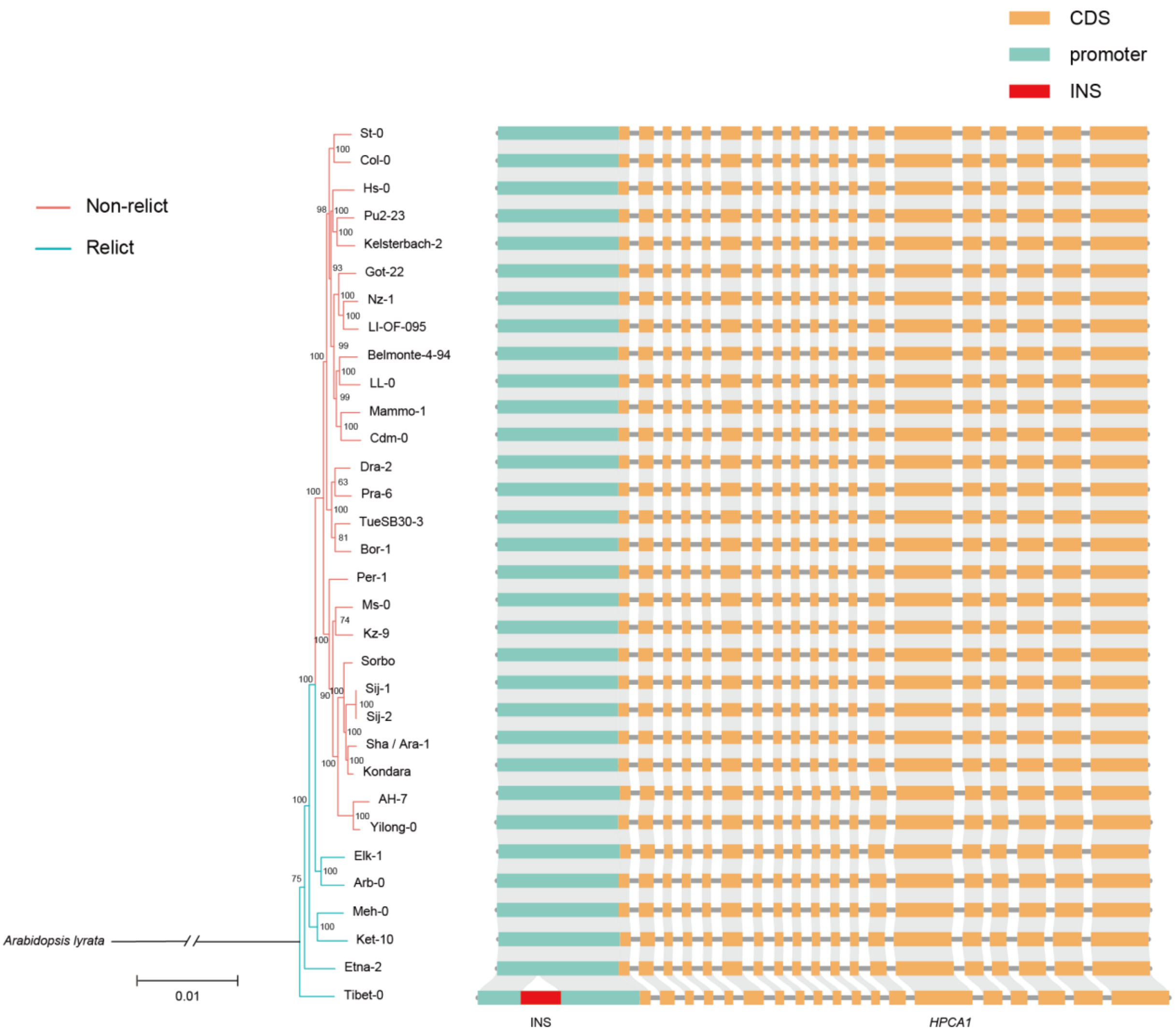

Supplementary Fig. 25. ***AT5G49760 (HPCA1)*** **gene structure in** ***Arabidopsis thaliana*** **ecotypes genomes.** Only Tibet-0 have a 332 bp TE insertion in the promoter region.

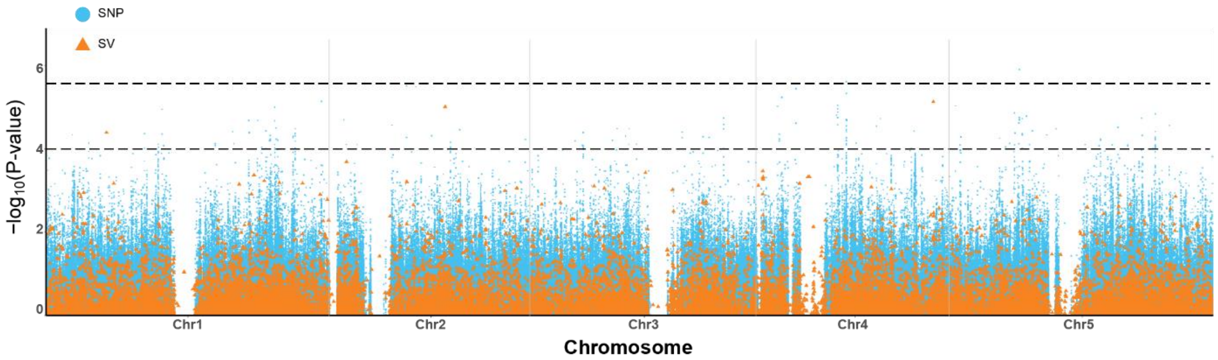

Supplementary Fig. 26. Manhattan plot of SV-GWAS (orange) and SNP-GWAS (blue) for Leaf As75 traits with detected association peaks. The dashed black lines were genome wide significance threshold for SNP-GWAS (upper, 5.80) and SV-GWAS (lower, 4.17). Significance was tested by standard linear mixed model.

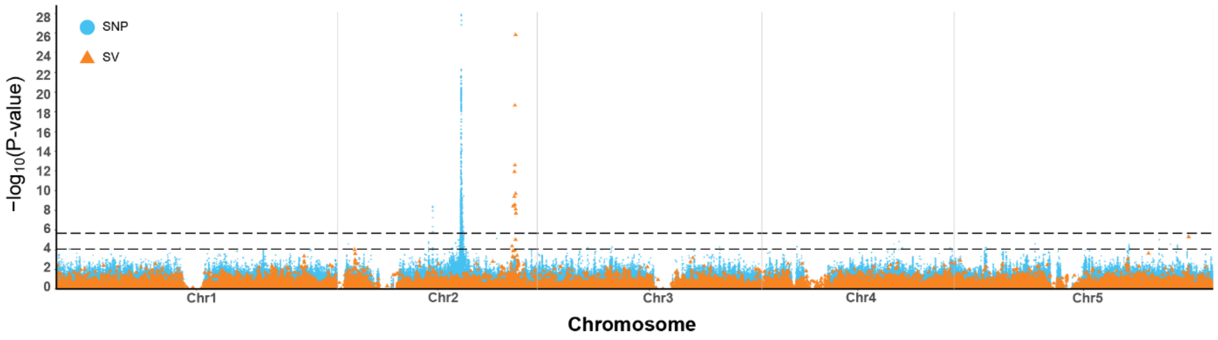

Supplementary Fig. 27. Manhattan plot of SV-GWAS (orange) and SNP-GWAS (blue) for Leaf Mo98 traits with detected association peaks. The dashed black lines were genome wide significance threshold for SNP-GWAS (upper, 5.80) and SV-GWAS (lower, 4.17). Significance was tested by standard linear mixed model.

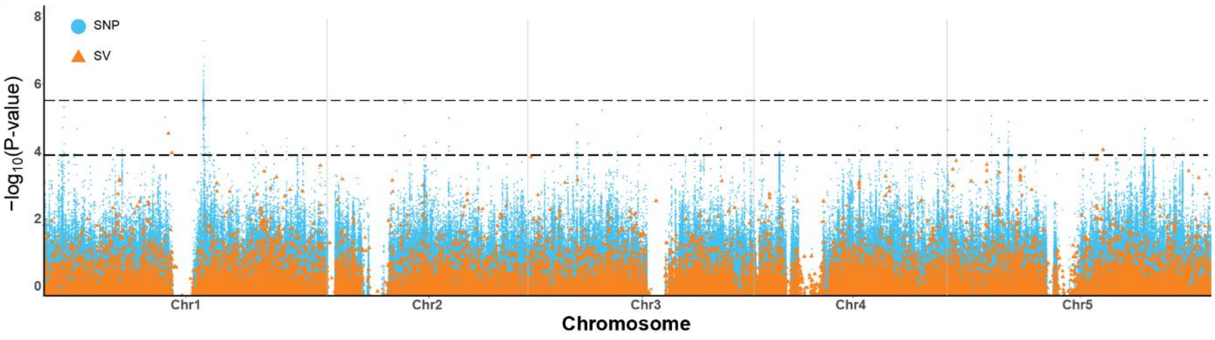

Supplementary Fig. 28. Manhattan plot of SV-GWAS (orange) and SNP-GWAS (blue) for Leaf P31 traits with detected association peaks. The dashed black lines were genome wide significance threshold for SNP-GWAS (upper, 5.80) and SV-GWAS (lower, 4.17). Significance was tested by standard linear mixed model.

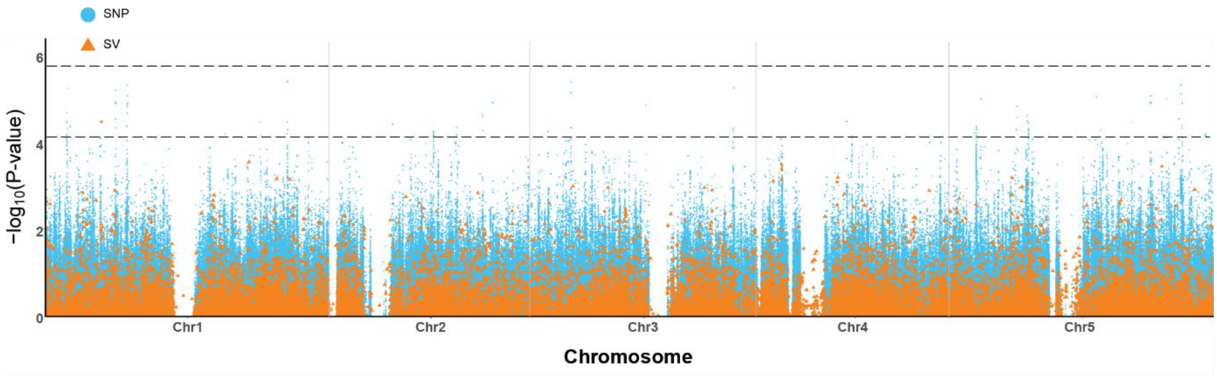

Supplementary Fig. 29. Manhattan plot of SV-GWAS (orange) and SNP-GWAS (blue) for Leaf S34 traits with detected association peaks. The dashed black lines were genome wide significance threshold for SNP-GWAS (upper, 5.80) and SV-GWAS (lower, 4.17). Significance was tested by standard linear mixed model.

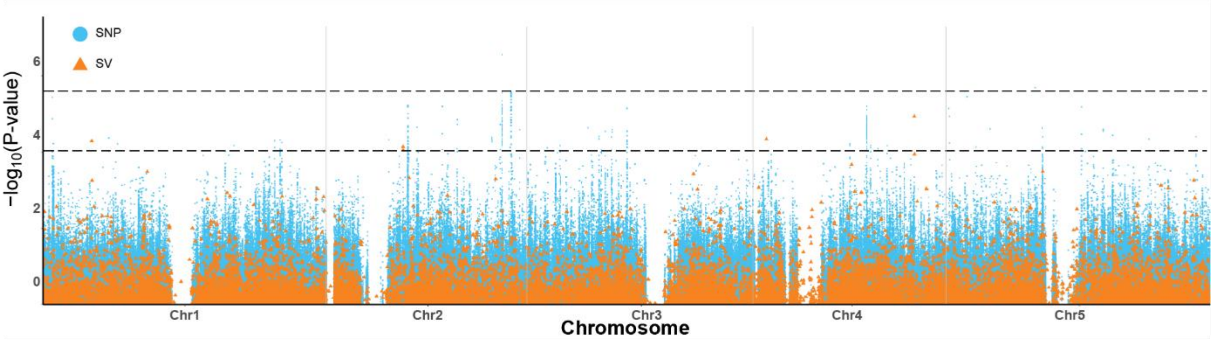

Supplementary Fig. 30. Manhattan plot of SV-GWAS (orange) and SNP-GWAS (blue) for Leaf Sr88 traits with detected association peaks. The dashed black lines were genome wide significance threshold for SNP-GWAS (upper, 5.80) and SV-GWAS (lower, 4.17). Significance was tested by standard linear mixed model.

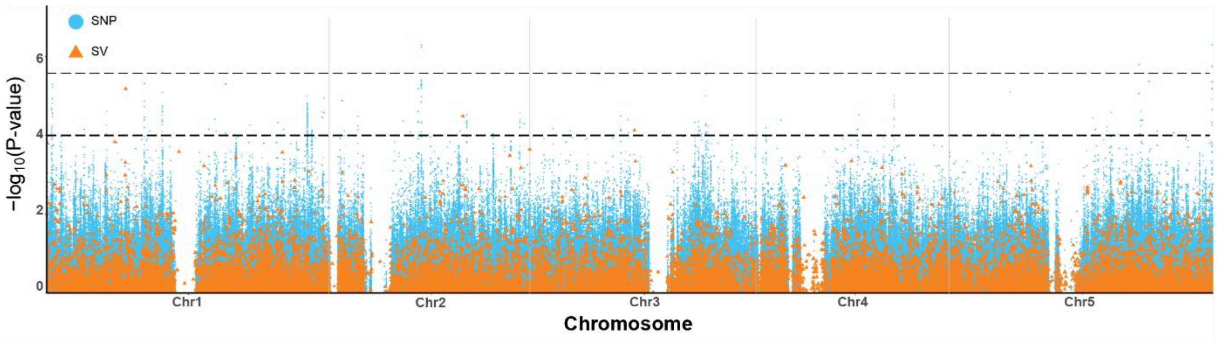

Supplementary Fig. 31. Manhattan plot of SV-GWAS (orange) and SNP-GWAS (blue) for Seed Ca43 traits with detected association peaks. The dashed black lines were genome wide significance threshold for SNP-GWAS (upper, 5.80) and SV-GWAS (lower, 4.17). Significance was tested by standard linear mixed model.

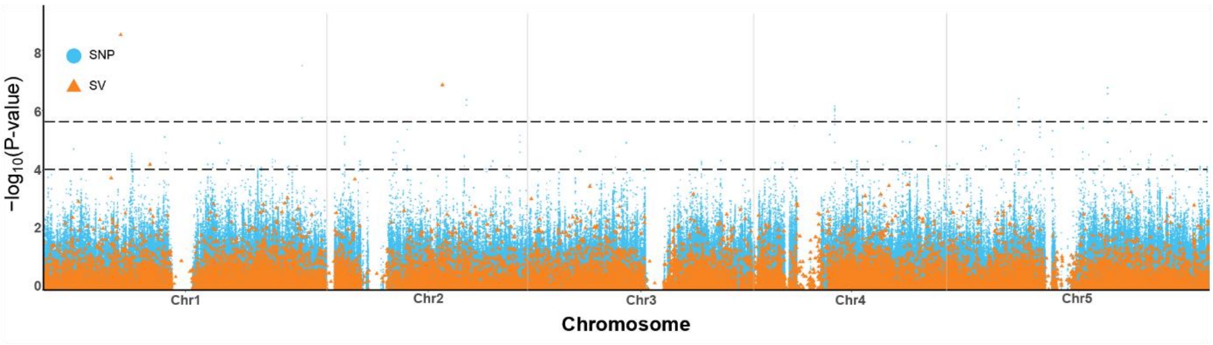

Supplementary Fig. 32. Manhattan plot of SV-GWAS (orange) and SNP-GWAS (blue) for Seed Cd114 traits with detected association peaks. The dashed black lines were genome wide significance threshold for SNP-GWAS (upper, 5.80) and SV-GWAS (lower, 4.17). Significance was tested by standard linear mixed model.

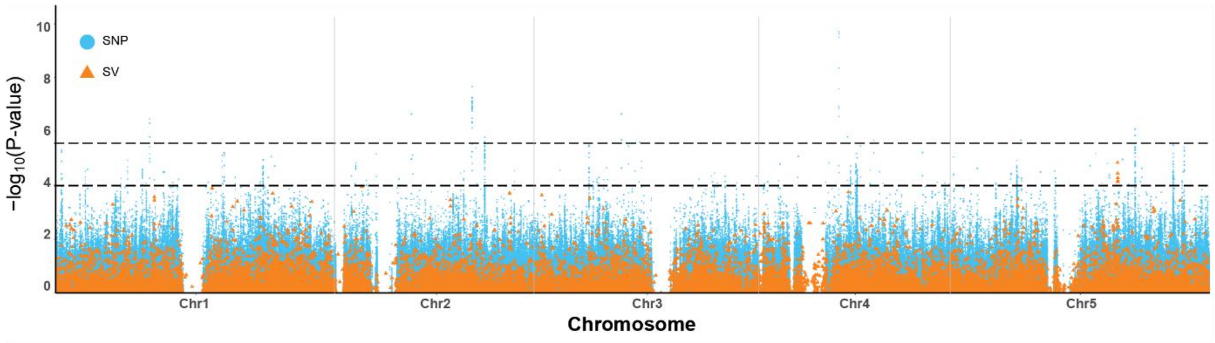

Supplementary Fig. 33. Manhattan plot of SV-GWAS (orange) and SNP-GWAS (blue) for Seed Mg25 traits with detected association peaks. The dashed black lines were genome wide significance threshold for SNP-GWAS (upper, 5.80) and SV-GWAS (lower, 4.17). Significance was tested by standard linear mixed model.

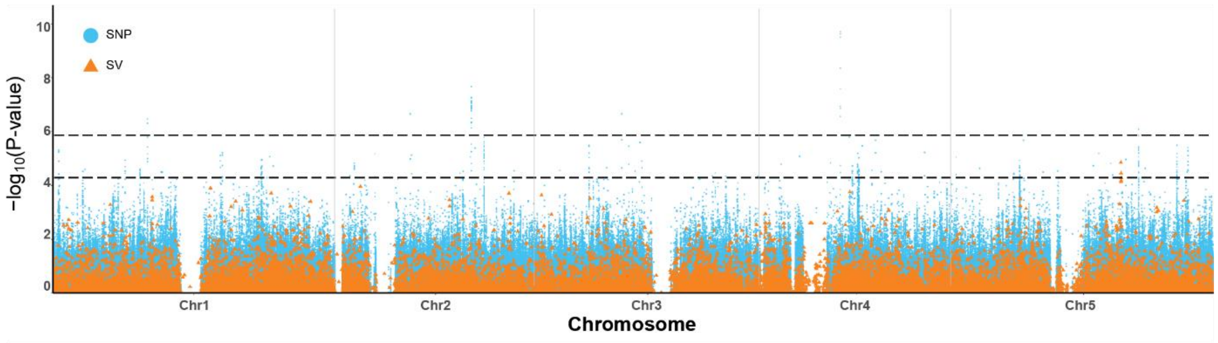

Supplementary Fig. 34. Manhattan plot of SV-GWAS (orange) and SNP-GWAS (blue) for Seed Mn55 traits with detected association peaks. The dashed black lines were genome wide significance threshold for SNP-GWAS (upper, 5.80) and SV-GWAS (lower, 4.17). Significance was tested by standard linear mixed model.

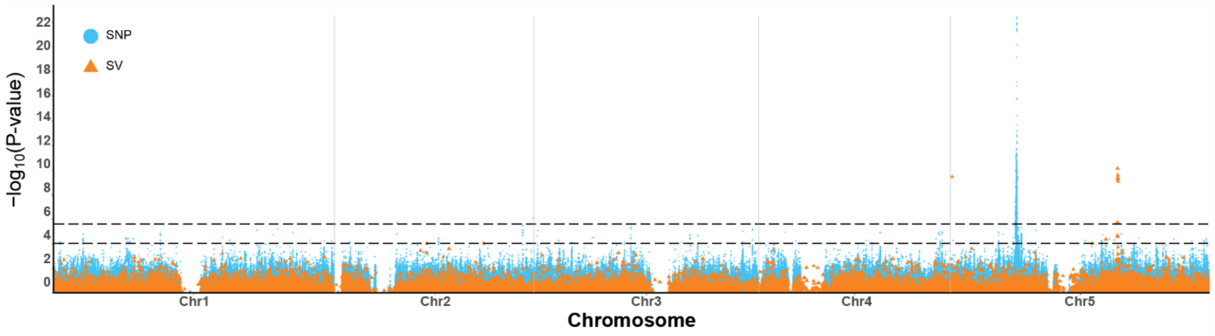

Supplementary Fig. 35. Manhattan plot of SV-GWAS (orange) and SNP-GWAS (blue) for Seed Se82 traits with detected association peaks. The dashed black lines were genome wide significance threshold for SNP-GWAS (upper, 5.80) and SV-GWAS (lower, 4.17). Significance was tested by standard linear mixed model.

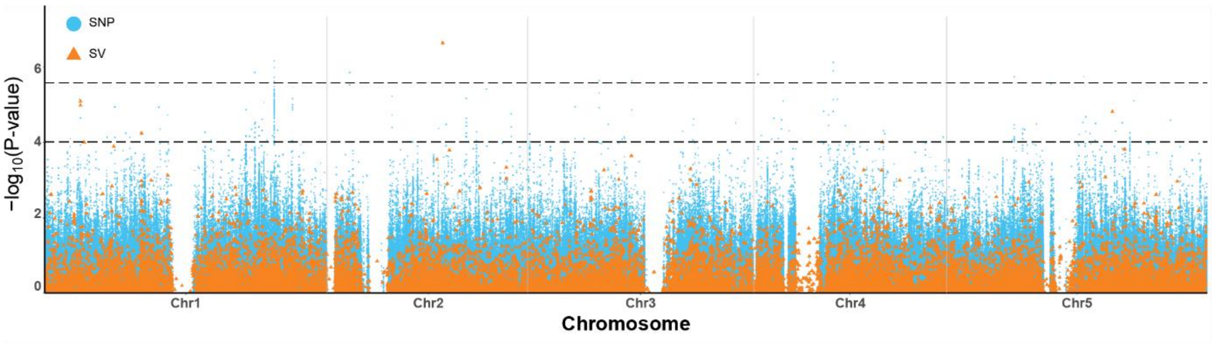

Supplementary Fig. 36. Manhattan plot of SV-GWAS (orange) and SNP-GWAS (blue) for Seed Zn66 traits with detected association peaks. The dashed black lines were genome wide significance threshold for SNP-GWAS (upper, 5.80) and SV-GWAS (lower, 4.17). Significance was tested by standard linear mixed model.
