# Supplementary material for: The pan-genome and local adaptation of Arabidopsis thaliana
Source: Nat Commun. 2023 Oct 6;14:6259. doi: 10.1038/s41467-023-42029-4 (PMC10558531; doi:10.1038/s41467-023-42029-4)
Supplement: Supplementary file 3 — Description of Additional Supplementary Files [file 41467_2023_42029_MOESM3_ESM.pdf]

Description of Additional Supplementary Files

File Name: Supplementary Data 1
Description: Variance component analysis for 61 traits with SNPs and SVs.

File Name: Supplementary Data 2
Description: Summary of the SV-GWAS results and genes around 20kb. Significance was tested by standard linear mixed model.

File Name: Supplementary Data 3
Description: Primers used in the LUC experiment.
