# Supplementary material for: The pan-genome and local adaptation of Arabidopsis thaliana
Source: Nat Commun. 2023 Oct 6;14:6259. doi: 10.1038/s41467-023-42029-4 (PMC10558531; doi:10.1038/s41467-023-42029-4)
Supplement: Supplementary file 7 — Reporting Summary [file 41467_2023_42029_MOESM7_ESM.pdf]

nature portfolio

Corresponding author(s): Jianquan Liu

Last updated by author(s): Sep 23, 2023

Reporting Summary

Nature Portfolio wishes to improve the reproducibility of the work that we publish. This form provides structure for consistency and transparency in reporting. For further information on Nature Portfolio policies, see our Editorial Policies and the Editorial Policy Checklist.

Statistics

For all statistical analyses, confirm that the following items are present in the figure legend, table legend, main text, or Methods section.

n/a	Confirmed	
☐	☒	The exact sample size (*n*) for each experimental group/condition, given as a discrete number and unit of measurement
☒	☐	A statement on whether measurements were taken from distinct samples or whether the same sample was measured repeatedly
☐	☒	The statistical test(s) used AND whether they are one- or two-sided<br>*Only common tests should be described solely by name; describe more complex techniques in the Methods section.*
☒	☐	A description of all covariates tested
☐	☒	A description of any assumptions or corrections, such as tests of normality and adjustment for multiple comparisons
☐	☒	A full description of the statistical parameters including central tendency (e.g. means) or other basic estimates (e.g. regression coefficient) AND variation (e.g. standard deviation) or associated estimates of uncertainty (e.g. confidence intervals)
☐	☒	For null hypothesis testing, the test statistic (e.g. *F*, *t*, *r*) with confidence intervals, effect sizes, degrees of freedom and *P* value noted<br>*Give P values as exact values whenever suitable.*
☒	☐	For Bayesian analysis, information on the choice of priors and Markov chain Monte Carlo settings
☒	☐	For hierarchical and complex designs, identification of the appropriate level for tests and full reporting of outcomes
☐	☒	Estimates of effect sizes (e.g. Cohen's *d*, Pearson's *r*), indicating how they were calculated

Our web collection on statistics for biologists contains articles on many of the points above.

Software and code

Policy information about availability of computer code

Data collection	No software had been used for data collection
Data analysis	Software used are listed as follows: SMRTLink v 9.0, GenomeScope v 2.0, hifiasm v 0.18, RagTag v 2.1.0, BUSCO v.5.0.2, EDTA v 2.1.0, RepeatMasker v 4.1.2-p1, LTR_retriever v 2.8, HISAT2 v 2.1.1, StringTie v 2.1.4, PASA v.2.3.3, AUGUSTUS v 3.2.3, GeneWise v 2.4.1, EVM v 1.1.1, eggNOG-mapper v2, NCBI BLAST v 2.2.30+, OrthoFinder v 1, MAFFT v 7.490, Gblocks v 0.91b, IQ-TREE v 2.0.3, PanGP v 1.0.1, R v 4.1.2, Trimmomatic v 0.38, KaKs_Calculator v 2.0, minigraph v 1, gfatools v 1, MCScanX v1 ,Minimap2 v.2.16, BWA v0.7.17-r1188, SAMtools v1.3.1, GATK v4.2, GCTA v1.

For manuscripts utilizing custom algorithms or software that are central to the research but not yet described in published literature, software must be made available to editors and reviewers. We strongly encourage code deposition in a community repository (e.g. GitHub). See the Nature Portfolio guidelines for submitting code & software for further information.

Data

Policy information about availability of data

All manuscripts must include a data availability statement. This statement should provide the following information, where applicable:

- Accession codes, unique identifiers, or web links for publicly available datasets
- A description of any restrictions on data availability
- For clinical datasets or third party data, please ensure that the statement adheres to our policy

The raw sequencing data for the PacBio HiFi reads, RNA sequencing reads, and re-sequencing Illumina short reads have been deposited in the Genome Sequence Archive (GSA)75 database at the National Genomics Data Center, Beijing Institute of Genomics, Chinese Academy of Sciences / China National Center for Bioinformation under BioProject PRJCA012695. The genome assembly, genome annotation, pan-TE library, graph pan-genome, gene family and gene presence/absence matrices files have been deposited in the Figshare database (https://figshare.com/articles/dataset/32_ecotypes_Arabidopsis_thaliana_genomes_gene_annotation_pan-TE_library_graph_pan-genome_gene_family_and_gene_presence_absence_matrices_files_/21673895). Public RNA-seq data were downloaded from the NCBI SRA database under BioProject PRJNA187928, PRJEB15161, and PRJNA319904. 1,135 individuals re-sequencing data were downloaded from PRJNA273563. 19 BIOCLIM and SRTM elevation data used in this study were download from WorldClim v2.1 (www.worldclim.org). The global UV-B radiation data was download from https://www.ufz.de/gluv. Source data are provided with this paper.

Research involving human participants, their data, or biological material

Policy information about studies with human participants or human data. See also policy information about sex, gender (identity/presentation), and sexual orientation and race, ethnicity and racism.

Reporting on sex and gender	No human research in this study.
Reporting on race, ethnicity, or other socially relevant groupings	No human research in this study.
Population characteristics	No human research in this study.
Recruitment	No human research in this study.
Ethics oversight	No human research in this study.

Note that full information on the approval of the study protocol must also be provided in the manuscript.

Field-specific reporting

Please select the one below that is the best fit for your research. If you are not sure, read the appropriate sections before making your selection.

☒ Life sciences ☐ Behavioural & social sciences ☐ Ecological, evolutionary & environmental sciences

For a reference copy of the document with all sections, see nature.com/documents/nr-reporting-summary-flat.pdf

Life sciences study design

All studies must disclose on these points even when the disclosure is negative.

Sample size	A total representative 32 individuals including 32 ecotypes of Arabidopsis thaliana were sequenced by HiFi long-reads sequencing technology.
Data exclusions	During the function annotation and following comparative analysis, we chose the longest transcript to represent each gene. As our article does not study on alternative splicing.
Replication	The genome sequences were taken and sequenced with more than 15-60 fold coverage. And the RT-qPCR and Dual-LUC activity assays experiments are independently performed three times. All attempts at replication were successful.
Randomization	In this study, all samples were collected based on their phenotypic characteristics and kinships, excluding closely related individuals.
Blinding	Blinding is not applicable in our study because it does not involve subjects which receive different treatments.

Reporting for specific materials, systems and methods

We require information from authors about some types of materials, experimental systems and methods used in many studies. Here, indicate whether each material, system or method listed is relevant to your study. If you are not sure if a list item applies to your research, read the appropriate section before selecting a response.

Materials & experimental systems

n/a	Involved in the study	
☒	☐	Antibodies
☒	☐	Eukaryotic cell lines
☒	☐	Palaeontology and archaeology
☒	☐	Animals and other organisms
☒	☐	Clinical data
☒	☐	Dual use research of concern
☐	☒	Plants

Methods

n/a	Involved in the study	
☒	☐	ChIP-seq
☒	☐	Flow cytometry
☒	☐	MRI-based neuroimaging

Plants

Seed stocks	Seed materials of AH-7 ecotype are collected from the wild. The other 31 seed materials are collected from Arabidopsis Biological Resource Center (ABRC). Supplementary Table 1 provides descriptions of the names, sampling locations, and CS number for the 32 ecotypes.
Novel plant genotypes	No novel plant genotypes were produced.
Authentication	N/A
